# Alchemical approach performance in calculating the ligand-binding free energy†

**DOI:** 10.1039/d4ra00692e

**Published:** 2024-05-08

**Authors:** Son Tung Ngo, Quynh Mai Thai, Trung Hai Nguyen, Nguyen Ngoc Tuan, T. Ngoc Han Pham, Huong T. T. Phung, Duong Tuan Quang

**Affiliations:** a Laboratory of Biophysics, Institute for Advanced Study in Technology, Ton Duc Thang University Ho Chi Minh City Vietnam ngosontung@tdtu.edu.vn; b Faculty of Pharmacy, Ton Duc Thang University Ho Chi Minh City Vietnam; c Faculty of Applied Sciences, Ton Duc Thang University Ho Chi Minh City Vietnam; d NTT Hi-Tech Institute, Nguyen Tat Thanh University Ho Chi Minh City Vietnam; e Department of Chemistry, Hue University Hue City Thua Thien Hue Province Vietnam dtquang@hueui.edu.vn

## Abstract

Alchemical binding free energy calculations are one of the most accurate methods for estimating ligand-binding affinity. Assessing the accuracy of the approach over protein targets is one of the most interesting issues. The free energy difference of binding between a protein and a ligand was calculated *via* the alchemical approach. The alchemical approach exhibits satisfactory accuracy over four targets, including AmpC beta-lactamase (AmpC); glutamate receptor, ionotropic kainate 1 (GluK1); heat shock protein 90 (Hsp90); and severe acute respiratory syndrome coronavirus 2 (SARS-CoV-2) main protease (Mpro). In particular, the correlation coefficients between calculated binding free energies and the respective experiments over four targets range from 0.56 to 0.86. The affinity computed *via* free energy perturbation (FEP) simulations is overestimated over the experimental value. Particularly, the electrostatic interaction free energy rules the binding process of ligands to AmpC and GluK1. However, the van der Waals (vdW) interaction free energy plays an important role in the ligand-binding processes of HSP90 and SARS-CoV-2 Mpro. The obtained results associate with the hydrophilic or hydrophobic properties of the ligands. This observation may enhance computer-aided drug design.

## Abstract 2

FEP calculation performance depends on the considered targets.

## Graphical abstract

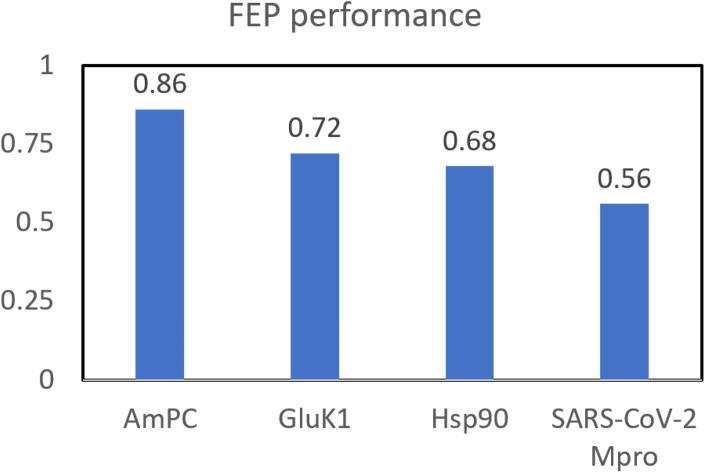

## Introduction

Binding processes between biomolecules are crucial in physical chemistry and related fields.^1^ Fundamental problems are associated with chemical reactions, including the interaction of a gene with another gene,^2^ of a gene with a protein,^3^ of a protein with another protein,^4^ and of a compound with a protein,^5^*etc.* Among these, noncovalent binding free energy, Δ*G*_bind_, can be computed *via* molecular dynamics (MD) simulations.^6^ The metric is related to the inhibition constant, *k*_i_, *via* the formula Δ*G*_bind_ = *RT* ln(*k*_i_), where *T* is the absolute temperature and *R* is the gas constant.

In computer-aided drug design (CADD), accurately estimating the value of Δ*G*_bind_ is a crucial task owing to its association with the protein–ligand binding mechanism.^6–10^ Moreover, the performance of the estimation of ligand-binding affinity relates to the reduction of therapeutic cost.^11,12^ Therefore, scientists have developed several computational methods to characterize ligand-binding free energies using physics as well as knowledge-based methods.^13–29^ Generally, the more accurate and precise the approach, the more the cost of computing resources.^30–33^ Among these, the free energy perturbation method,^34^ which is also known as the alchemical approach, is recognized to be one of the most accurate methods for determining ligand-binding affinity till date.^1,35^ However, the computational approach forms the correlation coefficient, which varies in a large range.^36,37^ Additionally, assessing the performance of a computational approach across different targets is interesting.^28,29,38–40^ More details, performance of AutoDock Vina over 800 experimental complexes were benchmarked.^39^ The SQM/COSMO filter scoring over various protein targets including acetylcholine esterase, TNF-α converting enzyme, aldose reductase, HIV-1 protease, and carbonic anhydrase II was tested.^28,29^ In these works, the SQM/COSMO filter scoring dominated over various approaches.

Recently, several studies were attempted to enhance the performance of alchemical calculations.^40–43^ However, overall, all of these schemes require massive costs of computing resources compared with the traditional ones. The combined approaches are less popular than the original FEP method. FEP calculations estimates absolute binding free energy between two biomolecules *via* two states without reference to a third state.^44–46^ FEP simulations are also performed to simply calculate the relative binding free energy between two biomolecules upon two states relative to a third state, which is typically the ground state.^47–49^ In addition, the performance of a computational approach strongly depend on applied targets,^28,29,50^ FEP performance over considered proteins is required to access.

Herein, the performance of the alchemical approach on 4 protein targets including AmpC beta-lactamase (AmpC), glutamate receptor, ionotropic kainate 1 (GluK1), heat shock protein 90 (Hsp90), and SARS-CoV-2 Mpro was assessed. The targets included AmpC involving 1XGI,^51^1XGJ,^51^2HDU,^52^2PU2,^53^2R9W,^53^2R9X,^53^3GR2,^54^4KZ3,^55^4KZ5,^55^ and 4OKP;^56^ GluK1 involving 1VSO,^57^2PBW,^57^2ZNS,^58^2ZNU,^58^3FV1,^58^ 3FV2,^58^3FVG,^58^3FVK,^58^4E0X,^59^ and 4DLD;^60^ Hsp90 involving 2QG0,^61^2QG2,^61^3K97,^62^3NMQ,^63^3QDD,^64^ 3 R4M,^65^4CWF,^66^4CWT,^66^4NH8,^67^ and 4O07;^68^ and SARS-CoV-2 Mpro involving 6M2N,^69^6WTT,^70^6XBG,^71^6XMK,^72^7B3E,^73^7L8I,^74^7LDL,^75^7NG3,^76^ 7CBT,^77^ and 8A4T.^78^ Alchemical calculations yielded an appropriate result for the three targets including AmpC, GluK1, and Hsp90. Moreover, applying FEP on SARS-CoV-2 Mpro requires additional calibrate.

## Material and methods

### Protein–ligand complexes

**Fig. 1:**
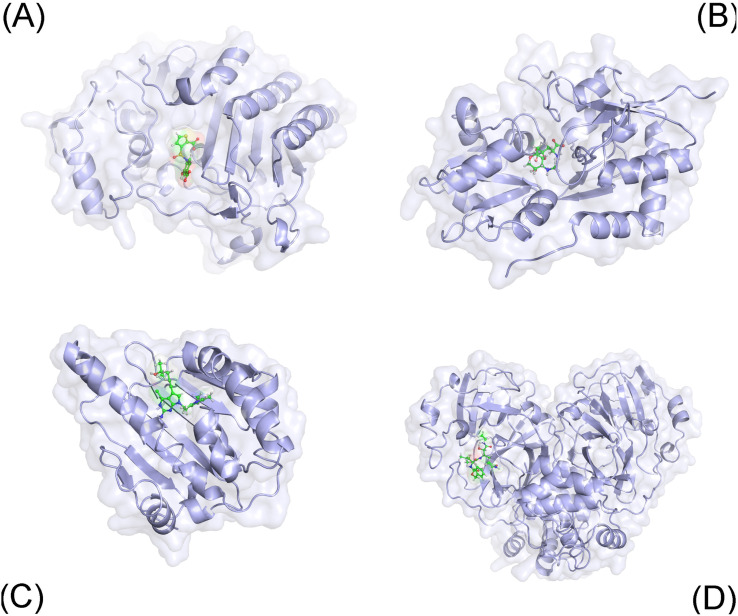
Initial conformation of considered systems including AmpC (A), GluK1 (B), Hsp90 (C), and SARS-CoV-2 Mpro (D).

The three-dimensional structure of 40 complexes (*cf.*Fig. 1 and Table S1 of the ESI†), whose identifies are expressed below, was obtained from the Protein Data Bank. Among these, proteins were parameterized *via* the Amber99SB-ILDN force field.^79^ The ligands were topologized by the general Amber force field.^80^ In particular, the charge of the ligands was achieved *via* the restrained electrostatic potential scheme^81^ using the quantum calculation at the B3LYP/6-31G(d,p) level of theory. Notably, the ligand protonation state was estimated *via* the chemicalize webserver.^82^ During simulations, the complex and individual ligand were inserted into a solvated box, which water molecule was parameterized *via* the TIP3P water model.^83^ Besides, the covalent binding between SARS-CoV-2 Mpro and ligand was not modelled in traditional MD simulations.

### Alchemical calculations

The perturbation simulations were performed to change the system from state A (bound) to B (unbound).^34^ In particular, a coupling parameter *λ* is typically used to conduct the task, and the process is known as perturbation simulations. The free energy change, Δ*G*, from different states was obtained *via* several perturbation simulations. Several values of *λ*, which include two sets of *λ*_elec_ and *λ*_vdW_ changing electrostatic and vdW interactions, respectively, ranging from 0 to 1 can be used to turn the Hamiltonian of the system from state A to B. Thus, Δ*G* can be computed *via* formula as follows1
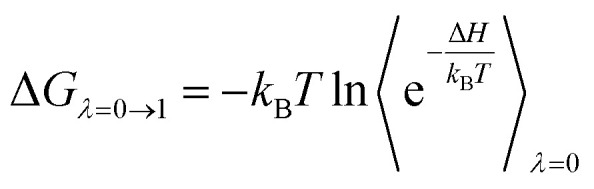
where *H* is the systemic Hamiltonian, *k*_B_ is the Boltzmann constant, and *T* is the absolute temperature. The Bennett acceptance ratio (BAR) method^84^ is employed to calculate the value.

The binding free energy between two biomolecules, Δ*G*_FEP_, can thus be calculated as follows

2 Δ*G*_FEP_ = Δ*G*_3_ − Δ*G*_1_

where Δ*G*_3_ and Δ*G*_1_ are the free energy changes between two states A and B of the protein–ligand in solution and ligand in solution (*cf.* Fig. S1 of the ESI†), respectively.

### Molecular dynamics simulations

GROMACS version 2019.6 with the general-purpose graphics processing unit acceleration was performed to simulate the complex and isolated ligand in solution systems.^85^ During the simulation, the periodic boundary condition was used on both systems. The MD simulation parameters were chosen referring to the previous work. In particular, the time step was selected as 0.002 ps during the Langevin dynamics simulations. The nonbond pair with a cut-off of 0.9 nm was refreshed every 10 steps of each integration. The particle-mesh Ewald method^86^ was used to present the electrostatic interaction at a cut-off of 0.9 nm. A cut-off scheme with a radius of 0.9 nm was utilized to treat the van der Waals (vdW) interaction.

The protein–ligand system in the solution was energy minimized using the steepest descent method. The simulations, which used NVT and NPT ensembles at 310 K with a length of 100 ps each simulation, were subsequently performed to relax the system. The C_α_ protein atoms were positionally fixed during NVT simulations using a harmonic potential. The solvated protein–ligand complex and free ligand in solution were simulated over 50 and 5 ns of MD simulations, respectively. The final conformations of both systems were used as the initial shape of perturbation simulations. The simulations were repeated two times.

The systemic Hamiltonian was modified over perturbation simulations under constant pressure conditions according to previous works.^87^ Among these, the electrostatic interaction between an inhibitor and surrounding atoms was changed through eight values of *λ*_elec_, including 0.00, 0.10, 0.20, 0.35, 0.50, 0.65, 0.80, and 1.00. The vdW interaction was modified *via* nine values of *λ*_vdW_ concerning 0.00, 0.10, 0.25, 0.35, 0.50, 0.65, 0.75, 0.90, and 1.00. The coupling parameters were chosen referring to the previous work.^8,87^ Each perturbation simulation had a length of 3.0 ns. The free energy changes, which include Δ*G*_3_ and Δ*G*_1_, were thus computed *via* the BAR method.^84^ The binding free energy of an inhibitor to a receptor was determined according to eqn (2).

### Analysis tools

Because ligand protonation states are crucial in the protein–ligand binding process,^88^ chemicalize webserver, a ChemAxon tool, was employed to predict ligand protonation states. The correlation coefficient error and the root-mean-square error (RMSE) were estimated using 1 000 000 rounds of the bootstrapping method.^89^ Sidechain (SC) contact between a ligand and an individual residue of a receptor was counted when the pair between nonhydrogen atoms of two groups is <4.5 Å. The hydrogen bond (HB) contact between a ligand and an individual residue of a receptor was measured when the angle donor–hydrogen–acceptor is >135° and the spacing between donors and acceptors is <3.5 Å. All-atom root-mean-square deviation (RMSD) of the complex and ligand systems was estimated *via* the GROMACS tools “gmx rms”. The experimental binding free energy, Δ*G*_EXP_, was calculated according to the formula

3 Δ*G*_EXP_ = *RT* ln(*k*_i_)

or

4 Δ*G*_EXP_ = *RT* ln(IC_50_),

where *R* is the gas constant, *T* is the absolute temperature, *k*_i_ is the inhibition constant, and IC_50_ is the half-maximal inhibitory concentration.

## Results and discussion

Assessing the performance of a computational approach is of useful as it may guide the following researchers who will consider the investigated targets.^90–94^ In particular, the benchmarking of the ability of a method depending on the targets probably has a large influence on the effectiveness and timeliness of the study.^95–97^ Further, although the alchemical approach is known as one of the most accurate protocols for determining the ligand-binding affinity, the obtained correlation coefficient varies in a large range.^36,37^ Herein, the accuracy of the alchemical calculation was thus evaluated over six targets included AmpC, GluK1, Hsp90, and SARS-CoV-2 Mpro.

Initially, conventional MD simulations were performed to turn the solvated system to stable states. Because the complexed structure was achieved from the experiments, the system rapidly reached equilibrium states (*cf.* Tables S2–S5 of the ESI†). In particular, the all-atom RMSD of the complex and ligand systems arrives at the stable regions after *ca.* 25.0 and 2.5 ns of MD simulations, respectively. Two replicas of each system were generated to reduce the error in the computation.

SC and HB contacts between protein–ligand complexes were analyzed over an interval of 25–50 ns of MD simulations, which corresponds to the equilibrium region. Results provide physical insights into the binding process of a ligand to a receptor. The results are shown in Fig. S2–S5 of the ESI.† By counting the residues formed more than rigid SC and HB contacts to inhibitors, the most influential factors controlling the inhibitor binding was clarified. The outcomes are described below.

The final shape of the systems was engaged as an input for free energy calculation *via* perturbation simulations. As mentioned above, the ligand-binding free energy to the receptor *via* the FEP approach, Δ*G*_FEP_, was calculated using the BAR method.^84^ Results indicated that FEP simulation is good in characterizing the ligand-binding free energy for the AmpC system. It is a good approach in estimating the ligand-binding affinity of three targets involving GluK1 and Hsp90. FEP calculations yield satisfactory results in the case of SARS-CoV-2. Further, the obtained correlation coefficients are described below. On average, the computed values, 〈Δ*G*_FEP_〉 = −14.62 ± 1.66 kcal mol^−1^, are overestimation compared with the respective experiments, 〈Δ*G*_EXP_〉 = −8.40 ± 0.34 kcal mol^−1^. Notably, 〈Δ*G*_FEP_〉 and 〈Δ*G*_EXP_〉 are the mean of calculated and experimental binding free energies over all of the considered systems. The overestimation of perturbation simulations is consistent with previous works.^33,98,99^ The uncorrected simulations of the interaction between ligands and surrounding molecules probably causes the different energy.^100,101^ Furthermore, the contributions of electrostatic, 〈Δ*G*_cou_〉, and vdW, 〈Δ*G*_vdW_〉, term to the binding free energy are −7.57 ± 1.85 and −7.06 ± 0.86 kcal mol^−1^, respectively. In addition, a good correlation coefficient between Δ*G*_FEP_ and Δ*G*_cou_, *R* = 0.88 ± 0.02, was found, confirming that electrostatic interaction plays an important role in the binding process of the considered systems.

### AmpC beta-lactamase

**Fig. 2:**
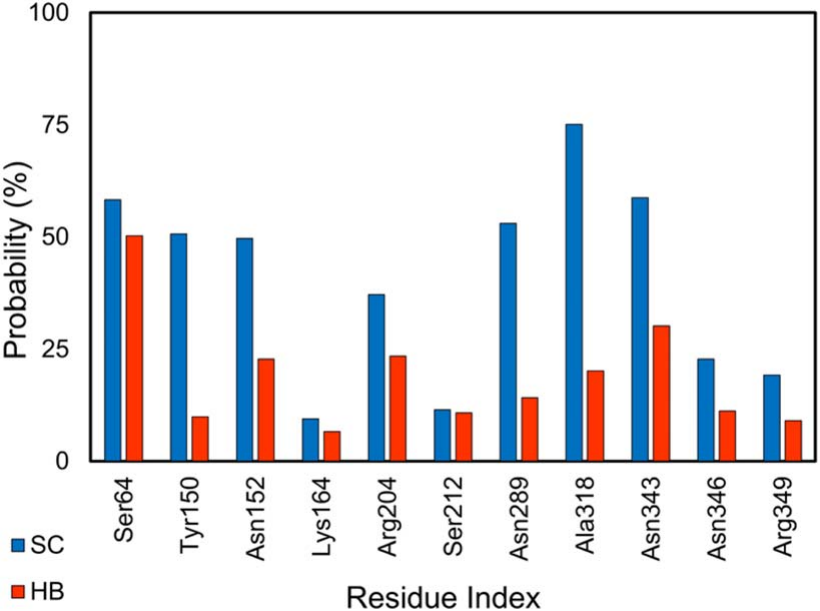
SC and HB contacts between essential residues of AmpC and inhibitors. The values were computed over an interval of 25–50 ns of MD simulations.

The enzyme clinically associates with the antibiotic resistance of Enterobacteriaceae and other organisms.^102^ Non-beta-lactam inhibitors of the AmpC have garnered considerable research interest.^51^ Several inhibitors for the AmpC have been developed.^51–53,55,56^ Using MD simulations, we gained physical insights into the binding process of these inhibitors to AmpC. Analyzing the equilibrium MD trajectories (*cf.* Table S2 of the ESI†) of these complexes, it was observed that residues *Ser64*, *Lys67*, *Tyr150*, *Asn152*, *Lys164*, *Arg204*, *Ser212*, *Asn289*, *Ala318*, *Asn343*, *Asn346*, and *Arg349* are important factors affecting the ligand-binding affinity because they rigidly adopt SC and HB contacts to inhibitors (*cf.*Fig. 2 and S2 of the ESI†). The outcomes are in good agreement with the previous work,^103^ especially the activity of *Lys67* and *Tyr150*, which are known as the catalytic residues, and *Ser64*, which is associated with the enzyme acyl adduct, would be thus inhibited. Therefore, probable mutations at these points could considerably alter the ligand-binding free energy to the AmpC.

**Fig. 3:**
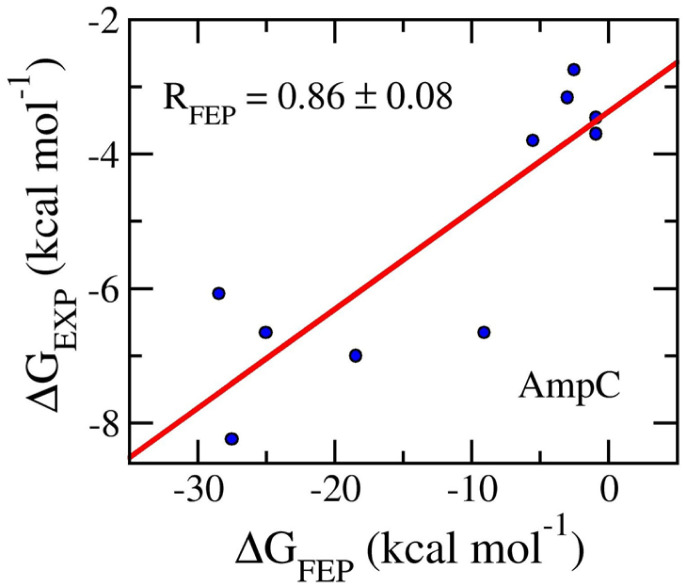
Correlation coefficients between calculated and experimental binding fee energy of AmpC systems. The computed value was obtained *via* FEP simulations.

The final snapshots of MD simulations were used as the starting shape of perturbation simulations. The work of the ligand decoupling process from the AmpC complexes in solution corresponds to the free energy change Δ*G*_3_ (*cf.* Fig. S1 of the ESI†). Further, the behavior of the ligand in the solution was mimicked. The Δ*G*_1_ value (*cf.* Fig. S1 of the ESI†) was thus calculated by estimating the work of the ligand decoupling process from the solvated ligand system. MD simulations involving the modified Hamiltonian were performed to obtain the work. The values of Δ*G*_3_ and Δ*G*_1_ were computed using the BAR method^84^ over an interval of 1–3 ns of the alter-*λ* simulations with every 100 ps. The obtained results are shown in Table 1. The calculated values of Δ*G*_FEP_ fall in the range from −0.90 ± 0.13 to −28.43 ± 0.48 kcal mol^−1^. The metric forms a good agreement with the respective experiments with an of *R*_AmpC_ = 0.86 ± 0.08 (*cf.*Fig. 3). It may be argued that FEP is a good approach to be able to rank the ligand-binding affinity for the AmpC target. Moreover, the means of electrostatic and vdW free energy difference of binding are −9.19 ± 3.44 and −2.95 ± 0.44 kcal mol^−1^, respectively. The outcome suggests that the AmpC inhibitors mostly are hydrophilic compounds. It is consistent with XLogP3 values, whose mean is 1.30 ± 0.51 (*cf.*Table 1). Furthermore, the average of computed metrics, 〈Δ*G*_FEP_〉 = −12.13 ± 3.45 kcal mol^−1^, is considerably smaller than that obtained *via* experiments, .*e.g.*, 〈Δ*G*_EXP_〉 = −5.15 ± 0.59 kcal mol^−1^. This means that the computed affinities are overestimated by a large value. The outcomes thus result in the large value of RMSE, which is of 11.67 ± 2.65 kcal mol^−1^. Although the large value of RMSE was obtained, the approach can be appropriately use for ranking the inhibitory of a ligand.

**Table 1:** Calculated *versus* experimental binding affinities between AmpC and its inhibitors

No.	PDB ID	XLogP3^a^	Δ*G*_cou_	Δ*G*_vdW_	Δ*G*_FEP_	Δ*G*_EXP_^b^
1	1XGI ^51^	1.8	−6.11	−2.97	−9.08 ± 0.77	−6.66 (ref. 51)
2	1XGJ ^51^	1.2	−23.84	−3.67	−27.51 ± 1.23	−8.24 (ref. 51)
3	2HDU ^52^	1.2	−0.19	−2.82	−3.01 ± 0.12	−3.16 (ref. 52)
4	2PU2 (ref. 53)	1.7	−25.69	−2.73	−28.43 ± 0.48	−6.08 (ref. 53)
5	2R9W ^53^	3.3	−17.55	−0.89	−18.44 ± 0.87	−7.00 (ref. 53)
6	2R9X ^53^	3.2	−20.63	−4.40	−25.03 ± 0.66	−6.66 (ref. 53)
7	3GR2 (ref. 54)	0.5	0.50	−1.41	−0.91 ± 2.60	−3.46 (ref. 54)
8	4KZ3 (ref. 55)	1.0	−2.53	−2.98	−5.51 ± 0.73	−3.80 (ref. 55)
9	4KZ5 (ref. 55)	1.9	−0.39	−2.12	−2.51 ± 0.48	−2.75 (ref. 55)
10	4OKP (ref. 56)	−2.8	4.58	−5.48	−0.90 ± 0.13	−3.70 (ref. 56)

a The values were obtained from the PubChem database.^104^

b The experimental binding free energies were computed based on the inhibition constant *k*_i_. The computed error was obtained using the bootstrapping method. The unit is kcal mol^−1^.

### Glutamate receptor, ionotropic kainate 1

**Fig. 4:**
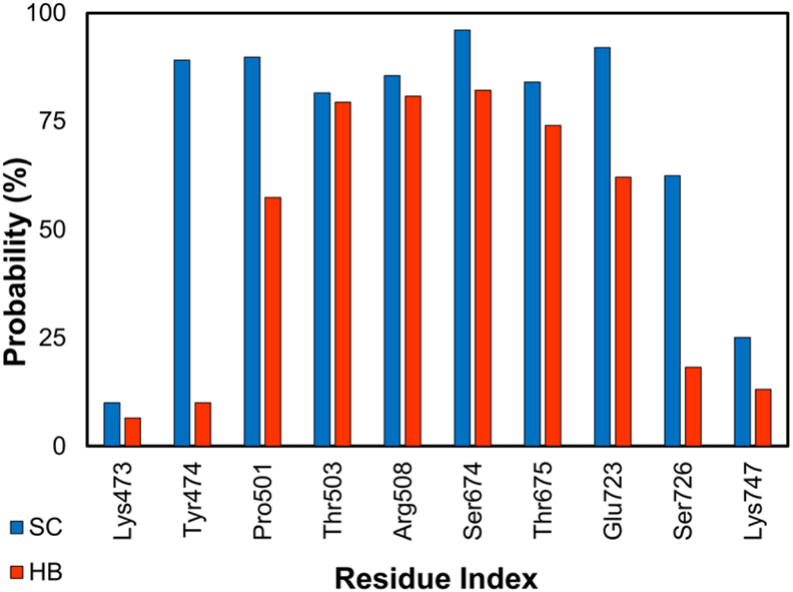
SC and HB contacts between essential residues of GluK1 and inhibitors. The values were computed over an interval of 25–50 ns of MD simulations of all complexes.

The GluK1 is a synaptic receptor that forms l-glutamate-gated ion channels and can play essential roles in excitatory neurotransmission of the nervous system.^57–60^ The GluK1 inhibition may deliver a defense against hyperexcitation.^57–60^ The ligand-binding domain (LBD) is a clamshell-shaped protein that makes a flexible conformation. LBD connects to the transmembrane domain of the GluK1 *via* a short linker.^105^ Ten LBD of the GluK1 + inhibitor systems were relaxed over MD simulations. The binding mechanism of the protein and its ligand is thus illuminated throughout analyzing the free energy difference of binding and SC/HB contacts. The all-atom RMSD of both complexes and ligands in solution systems are described in Table S2 of the ESI.† The residues involving *Lys473*, *Tyr474*, *Pro501*, *Thr503*, *Arg508*, *Ser674*, *Thr675*, *Glu723*, *Ser726*, and *Lys747* were found to be able to rigidly adopt SC and HB contacts with GluK1 (*cf.*Fig. 4 and S3 of the ESI†). Notably, 10 complexes were obtained from *Homo sapiens* and *Rattus norvegicus*; however, their sequence is almost identical (*cf.* the ESI†).

**Fig. 5:**
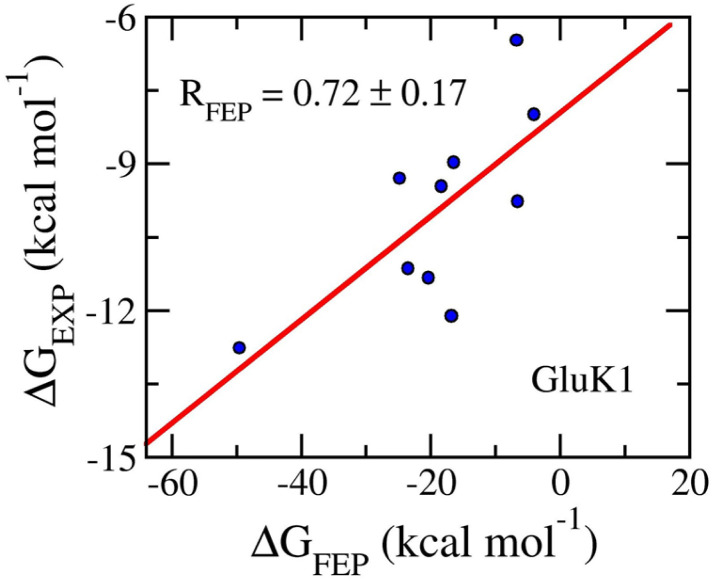
Correlation coefficients between calculated and experimental binding free energy of GluK1 systems. The computed value was obtained *via* FEP simulations.

Ten ligands were then annihilated from both complexes and ligands in solution systems *via* perturbation simulations. The BAR method^84^ was used to assess the free energy change during ligand annihilation. The binding free energy was obtained and is described in Table 2. The obtained results show that Δ*G*_FEP_ varies in the range from −49.54 ± 4.39 to −3.04 ± 0.32 kcal mol^−1^. Moreover, the calculated binding free energies procedure a correlation coefficient with the respective experiments by an of *R*_GluK1_ = 0.76 ± 0.13 (Fig. 5). The computed errors are RMSE_GluK1_ = 14.56 ± 4.25 kcal mol^−1^. Similar to the AmpC systems, the FEP approach is an appropriate protocol to rank the ligand-binding affinity, but the absolute values overestimate the experimental data. The overestimation may be caused by the inaccuracy interaction simulation of the force field as mentioned above.^100,101^ Furthermore, the means of electrostatic, 〈Δ*G*_cou_〉, and vdW, 〈Δ*G*_vdW_〉, free energy difference of binding are −16.23 ± 4.08 and −2.54 ± 0.48 kcal mol^−1^, respectively. The dominance of Δ*G*_cou_ over Δ*G*_vdW_ is in good agreement with the circumstance that GluK1 inhibitors are highly hydrophilic substances, 〈XLogP3〉 = −3.29 ± 0.55.

**Table 2:** Calculated *versus* experimental binding affinities between GluK1 and its inhibitors

No.	PDB ID	XLogP3^a^	Δ*G*_cou_	Δ*G*_vdW_	Δ*G*_FEP_	Δ*G*_EXP_^b^
1	1VSO ^57^	−2.7	−3.57	−3.15	−6.72 ± 0.95	−6.48 (ref. 57)
2	2PBW ^57^	−1.3	−18.56	−1.74	−20.3 ± 3.23	−11.33 (ref. 57)
3	2ZNS ^58^	−3.7	−15.68	−0.76	−16.44 ± 2.74	−8.97 (ref. 58)
4	2ZNU ^58^	−5.1	−20.03	−3.47	−23.50 ± 0.02	−11.14 (ref. 58)
5	3FV1 (ref. 58)	−7.1	−45.54	−4.00	−49.54 ± 4.39	−12.77 (ref. 58)
6	3FVN ^58^	−2.8	−23.25	−1.51	−24.76 ± 2.36	−9.30 (ref. 58)
7	3FVG ^58^	−3.1	−14.56	−3.77	−18.33 ± 0.23	−9.46 (ref. 58)
8	3FVK ^58^	−4.1	−12.78	−4.01	−16.79 ± 0.74	−12.11 (ref. 58)
9	4E0X ^59^	−1.8	−3.05	−3.51	−6.56 ± 2.58	−9.77 (ref. 59)
10	4DLD ^60^	−1.2	−0.20	−3.81	−4.01 ± 0.38	−7.99 (ref. 60)

a The values were obtained from the PubChem database.^104^

b The experimental binding free energies were computed based on the inhibition constant *k*_i_. The computed error was obtained using the bootstrapping method. The unit is kcal mol^−1^.

### Heat shock protein 90

**Fig. 6:**
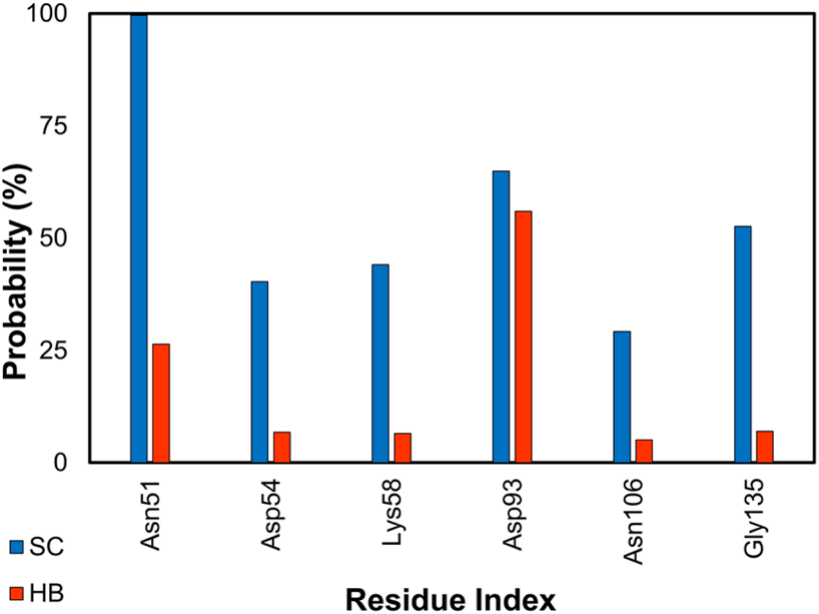
SC and HB contacts between essential residues of GluK1 and inhibitors. The values were computed over an interval of 25–50 ns of MD simulations.

Hsp90 is a highly conserved molecular chaperone protein found in all eukaryotic cells.^106^ Hsp90 acts as a chaperone for a large number of client proteins, participating in various cellular processes, such as signal transduction, cell cycle regulation, DNA repair, and apoptosis. It is crucial for the folding, maturation, and degradation of proteins. In addition to its normal functions, Hsp90 has been implicated in several disease processes, including cancer, neurodegenerative diseases, and infectious diseases, making it an attractive target for therapeutic intervention.^107,108^ Over the stable snapshots produced by MD simulations, essential residues considerably contribute to the ligand-binding affinity that can be estimated *via* contact analyses. Among these, the residues *Asn51*, *Asp93*, *Gly135*, *Phe138*, *Tyr139*, and *Thr184* form SC and HB contacts to inhibitors more than 50% and 5% of considered snapshots (*cf.*Fig. 6), respectively. This is in good agreement with previous studies.^62,109^

**Fig. 7:**
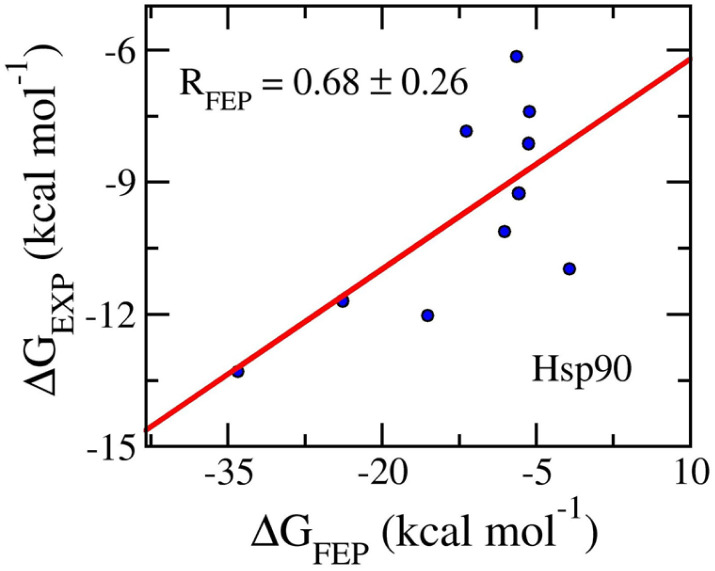
Correlation coefficients between calculated and experimental binding free energy of Hsp90 systems. The computed value was obtained *via* FEP simulations.

The ligand-binding free energy between Hsp90 and ligands was determined *via* FEP simulations^34^ and is shown in Table 3. The calculated binding free energy, Δ*G*_FEP_, which adopts in the range from −36.20 ± 1.09 to −5.62 ± 0.16 kcal mol^−1^, forms a good correlation to the corresponding experiments, *R*_Hsp90_ = 0.76 ± 0.15 (Fig. 7). The computed error is of RMSE_Hsp90_ = 8.52 ± 3.08 kcal mol^−1^. The obtained binding affinity overemphasizes the respective experiments, but the approach can be used for ranking ligand-binding affinity. The observation is consistent with previous mention. Moreover, the vdW interaction free energy, 〈Δ*G*_vdW_〉 = −13.21 ± 1.57 kcal mol^−1^, dominates over the electronic ones, 〈Δ*G*_cou_〉 = 0.35 ± 2.65 kcal mol^−1^. The dominant of Δ*G*_vdW_ is in good agreement with the fact that Hsp90 inhibitors are hydrophobic compounds, 〈XLogP3〉 = 2.56 ± 0.33. It may be argued that the binding mechanism of ligands to Hsp90 is considerably different from those of the AmpC and GluK1 systems.

**Table 3:** Calculated *versus* experimental binding affinities between Hsp90 and its inhibitors

No.	PDB ID	XLogP3^a^	Δ*G*_cou_	Δ*G*_vdW_	Δ*G*_FEP_	Δ*G*_EXP_^b^
1	2QG0 (ref. 61)	0.8	−1.80	−9.97	−11.78 ± 1.24	−7.85 (ref. 61)
2	2QG2 (ref. 61)	1.6	6.58	−12.20	−5.62 ± 0.16	−7.41 (ref. 61)
3	3K97 (ref. 62)	4.2	1.58	−3.30	−3.3 ± −1.72	−10.98 (ref. 62)
4	3NMQ ^63^	3.1	−18.04	−15.95	−15.95 ± −33.99	−13.31 (ref. 63)
5	3QDD ^64^	1.9	1.10	−16.64	−15.54 ± 0.36	−12.04 (ref. 64)
6	3 R4M^65^	2.1	−0.57	−5.13	−5.70 ± 0.64	−8.13 (ref. 65)
7	4CWF ^66^	3.0	4.17	−11.03	−6.86 ± 0.77	−6.16 (ref. 66)
8	4CWT ^66^	2.9	7.54	−14.22	−6.68 ± 0.40	−9.26 (ref. 66)
9	4NH8 (ref. 67)	3.2	−1.63	−22.18	−23.80 ± 0.16	−11.70 (ref. 67)
10	4O07 (ref. 68)	4.1	10.80	−18.85	−8.05 ± 1.65	−10.13 (ref. 68)

a The values were obtained from the PubChem database.^104^

b The experimental binding free energies were computed based on the inhibition constant *k*_i_. The computed error was obtained using the bootstrapping method. The unit is kcal mol^−1^.

### SARS-CoV-2 main protease

**Fig. 8:**
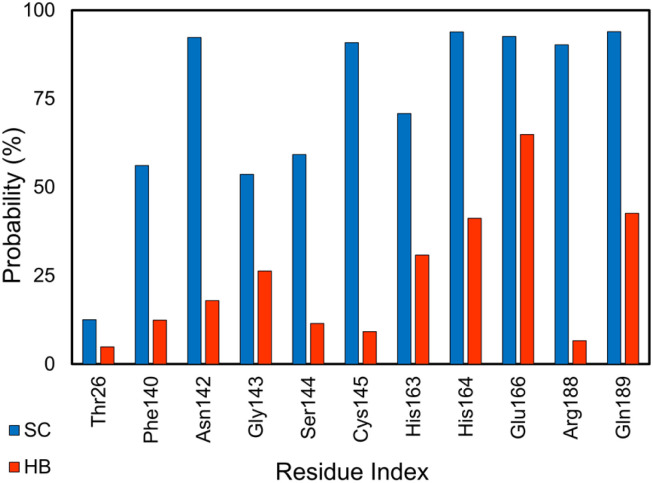
SC and HB contacts between essential residues of SARS-CoV-2 Mpro and inhibitors. The values were computed over an interval of 25–50 ns of MD simulations.

SARS-CoV-2 Mpro is an enzyme that plays a crucial role in the replication of SARS-CoV-2, which causes COVID-19.^110,111^ The main protease is responsible for cleaving large viral polyproteins into functional proteins that are essential for the virus to replicate and infect host cells.^112^ This makes the main protease an attractive target for drug development as inhibiting its activity could potentially stop the virus from replicating.^113^ Analyzing the HB and SC contacts between SARS-CoV-2 Mpro and its inhibitors, the residues *Thr26*, *Phe140*, *Asn142*, *Gly143*, *Ser144*, *Cys145*, *His163*, *His164*, *Met165*, *Glu166*, *Arg188*, and *Gln189* adopts more than 5% and 12% of HB and SC contacts over equilibrium snapshots, respectively (*cf.*Fig. 8). It is in good agreement with the work on the SARS-CoV-2 Mpro monomer.^87^

**Fig. 9:**
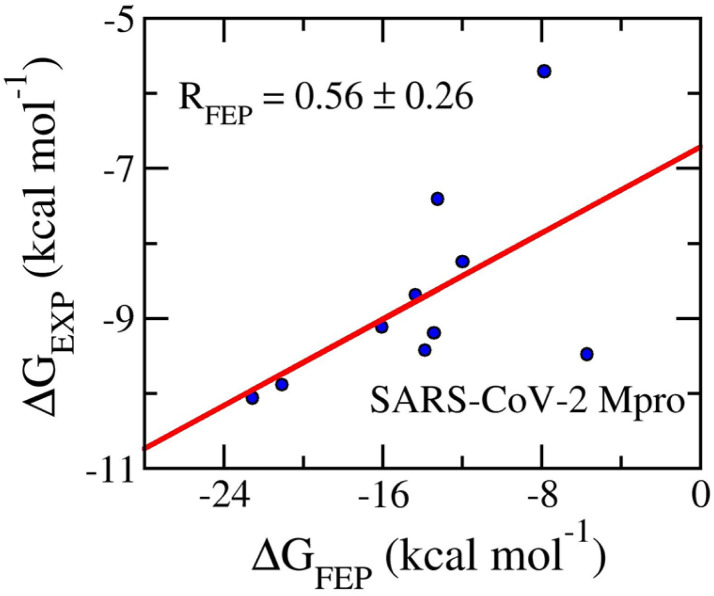
Correlation coefficients between calculated and experimental binding free energy of SARS-CoV-2 Mpro systems. The computed value was obtained *via* FEP simulations.

Table 4 shows the FEP binding free energy compared to the respective experiment and the value of the ligand XLogP3. The obtained value of Δ*G*_FEP_ falls in the range from −22.54 ± 3.52 to −5.69 ± 1.83 kcal mol^−1^. The calculated results form an appropriate correlation coefficient with the corresponding experiment by a value of *R*_Mpro_ = 0.56 ± 0.26 (Fig. 9). The inaccurate part may be caused by the fact that the conventional MD simulations do not mimic the covalent binding of ligands to the residue *Cys145* of SARS-CoV-2 Mpro.^114–116^ Moreover, in consistency with previous works, the SARS-CoV-2 Mpro inhibitors form an average of XLogP3 = 2.19 ± 0.30. It may be argued that the inhibitors will adopt a stronger vdW interaction free energy to the protease than electrostatic ones. Indeed, the mean of vdW interaction free energy, 〈Δ*G*_vdW_〉, is −9.11 ± 0.73 kcal mol^−1^, which is considerably larger than the mean of electrostatic interaction free energy, 〈Δ*G*_cou_〉, is of −4.89 ± 1.72 kcal mol^−1^. This is in good agreement with the previous study.^117^

**Table 4:** Calculated *versus* experimental binding affinities between SARS-CoV-2 Mpro and its inhibitors

No.	PDB ID	XLogP3^a^	Δ*G*_cou_	Δ*G*_vdW_	Δ*G*_FEP_	Δ*G*_EXP_^c^
1	6M2N ^69^ d	1.7	−4.36	−7.61	−11.97 ± 0.17	−8.27 (ref. 69)
2	6WTT ^70^ e	1.7^b^	−5.20	−8.21	−13.41 ± 1.76	−9.23 (ref. 111)
3	6XBG ^71^ e	1.6^b^	−10.08	−12.46	−22.54 ± 3.52	−10.08 (ref. 71)
4	6XMK ^72^ e	2.3^b^	−8.16	−6.18	−14.34 ± 0.38	−8.71 (ref. 72)
5	7B3E ^73^ e	1.2	−11.32	−4.71	−16.03 ± 2.07	−9.14 (ref. 118)
6	7L8I ^74^ d	3.2	2.47	−10.32	−7.85 ± 3.23	−5.72 (ref. 119)
7	7LDL ^75^ e	1.9	−11.02	−10.03	−21.05 ± 3.08	−9.91 (ref. 75)
8	7NG3 (ref. 76)^e^	4.6	−3.63	−9.58	−13.21 ± 1.96	−7.42 (ref. 70)
9	7LCR ^75^ e	1.7^b^	−3.68	−10.18	−13.86 ± 0.34	−9.43 (ref. 72)
10	8 A4T^78^^e^	2.0	6.09	−11.78	−5.69 ± 1.83	−9.50 (ref. 78)

a The values were obtained from the PubChem database.^104^

b The value was calculated *via* the XLogP3 server.^120^

c The experimental binding free energies were computed based on the inhibition constant IC_50_.

d The non-covalent binding ligands.

e The covalent binding ligands. The computed error was obtained using the bootstrapping method. The unit is kcal mol^−1^.

Overall, the calculated results are overestimation. The overestimation of perturbation simulations is discussed above as it is probably caused by the uncorrected interaction between ligands and surrounding molecules.^100,101^ Moreover, the net charge of complex and free inhibitor in solution are not equal to zero, the effects can be large and lead to increase RMSE. A correlation is thus required to be used to fix the overestimation due to the change of systemic net charge.^121^ Furthermore, four complexes including 1XGJ, 3FV1, 3K97, and 6M2N were randomly selected for FEP calculations (*cf.* Tables S6 and S7 and Fig. S6 of the ESI†) without consideration of the protonation states of ligands, which may be a hypothesis that it is equivalent to use coupled ions to neutralize the ligand charge. In this case, the average of binding free energy is of −12.02 ± 3.21 kcal mol^−1^ in comparison with −24.36 ± 1.62 kcal mol^−1^ of considering protonated ligands. It should be noted that the mean of Δ*G*_EXP_ is of −10.06 ± 0.96 kcal mol^−1^. The RMSE of systems with neutralized ligands, RMSE = 5.05 ± 2.47 kcal mol^−1^, is significantly smaller than the system with protonated ligands, RMSE = 20.88 ± 7.41 kcal mol^−1^. The calculated value of Δ*G*_FEP_, when neutralized ligands were considered, formed a correlation coefficient *R* = 0.94 compared with the respective experiments. The correlation coefficient, when protonated ligands were investigated, only is of *R* = 0.56. Absolutely, when neutralized ligands were considered, the correlation coefficient was significantly increased and the RMSE value was rigidly decreased. Further investigation to be able to clarify the issues would be carried out in the future.

## Conclusion

Herein, we have demonstrated that FEP calculation is a good approach to characterize the binding free energy of ligands to AmpC, GluK1, Hsp90, and SARS-CoV-2 Mpro.

The computed affinity *via* FEP simulations overestimates the corresponding experimental values. In particular, the electrostatic interaction free energy rules the ligand-binding to AmpC and GluK1. However, the van der Waals (vdW) interaction free energy plays an important role in the ligand-binding processes of HSP90 and SARS-CoV-2 Mpro. The outcomes associate with the hydrophilic or hydrophobic properties of the ligands. Moreover, the important residues controlling the binding process of ligands to four receptors were clarified *via* SC and HB analyses. The possible mutation at these points may alter the ligand-binding free energy, causing drug resistance. Furthermore, the accuracy of the FEP approach based on conventional MD simulations may be reduced when covalent binding ligands are considered. In addition, the net charge of complex and free inhibitor in solution are not equal to zero, the effects can be large and lead to increase RMSE. A correlation is thus required to be used to fix the overestimation due to the change of systemic net charge.^121^ A benchmark without consideration of the protonation states of ligands, which may be a hypothesis that it is equivalent to use coupled ions to neutralize the ligand charge, would be performed in the future as well as compared with the other methods such as MM-PBSA or LIE approaches.

## Conflicts of interest

There are no conficts to declare.

## Supplementary Material

RA-014-D4RA00692E-s001
