# Supplementary material for: Alchemical approach performance in calculating the ligand-binding free energy
Source: RSC Adv. 2024 May 8;14(21):14875–85. doi: 10.1039/d4ra00692e (PMC11078002; doi:10.1039/d4ra00692e)
Supplement: RA-014-D4RA00692E-s001 [file RA-014-D4RA00692E-s001.pdf]

Electronic Supplementary Material (ESI) for RSC Advances.
This journal is © The Royal Society of Chemistry 2024

**Supporting Information**

# Alchemical Approach Performance in Calculating the Ligand-Binding Free Energy

Son Tung Ngo,[ab*] Quynh Mai Thai,[ab] Trung Hai Nguyen,[ab] Nguyen Ngoc Tuan,[c] T. Ngoc Han Pham,[d] Huong T. T. Phung,[d] and Duong Tuan Quang[e*]

[a]*Laboratory of Biophysics, Institute for Advanced Study in Technology, Ton Duc Thang University, Ho Chi Minh City, Vietnam*

[b]*Faculty of Pharmacy, Ton Duc Thang University, Ho Chi Minh City, Vietnam*

[c]*Faculty of Applied Sciences, Ton Duc Thang University, Ho Chi Minh City, Vietnam*

[d]*NTT Hi-Tech Institute, Nguyen Tat Thanh University, Ho Chi Minh City, Vietnam*

[e]*Department of Chemistry, Hue University, Thua Thien Hue Province, Hue City, Vietnam*

*Email: ngosontung@tdtu.edu.vn and dtquang@hueuni.edu.vn

Sequence comparison between *Homo sapiens* and *Rattus norvegicus* of Glutamate Receptor, Ionotropic Kainate 1
Pairwise Alignment
Sequence 1: 2ZNS (*Homo sapiens*)
Sequence 2: 4DLD (*Rattus norvegicus*)
Sequence ends allowed to slide over each other
Alignment score: 248

Identities: 0.9766537
Similarities: 0.9766537
Similarity Matrix: MATCH

```
2ZNS  EANRTLIVTTILEEPYVMYRKSDKPLYGNDRFEGYCLDLLKELSNILGFIYDVKLVPDGKYGAQNDKGEWNGMVKELIDH
4DLD  --NRTLIVTTILEEPYVMYRKSDKPLYGNDRFEGYCLDLLKELSNILGFLYDVKLVPDGKYGAQNDKGEWNGMVKELIDH

2ZNS  RADLAVAPLTITYVREKVIDFSKPFMTLGISILYRKGTPIDSADDLAKQTKIEYGAVRDGSTMTFFKKSKISTYEKMWAF
4DLD  RADLAVAPLTITYVREKVIDFSKPFMTLGISILYRKGTPIDSADDLAKQTKIEYGAVRDGSTMTFFKKSKISTYEKMWAF

2ZNS  MSSRQQTALVRNSDEGIQRVLTTDYALLMESTSIEYVTQRNCNLTQIGGLIDSKGYGVGTPIGSPYRDKITIAILQLQEE
4DLD  MSSRQQSALVKNSDEGIQRVLTTDYALLMESTSIEYVTQRNCNLTQIGGLIDSKGYGVGTPIGSPYRDKITIAILQLQEE

2ZNS  GKLHMMKEKWWRGNGC-
4DLD  GKLHMMKEKWWRGNGCP
```

**Table S1.** 2D and 3D structure for all ligands of AmpC, GluK1, Hsp90 and SARS-CoV-2 Mpro systems

| $N^0$ | PDB ID | 2D Structure | 3D Structure |
|---|---|---|---|
| 1 | 1XGI | 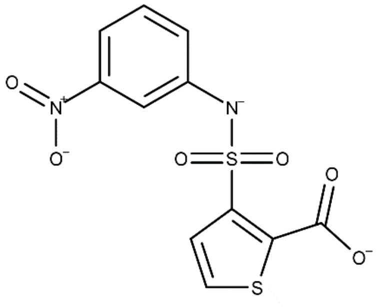 | 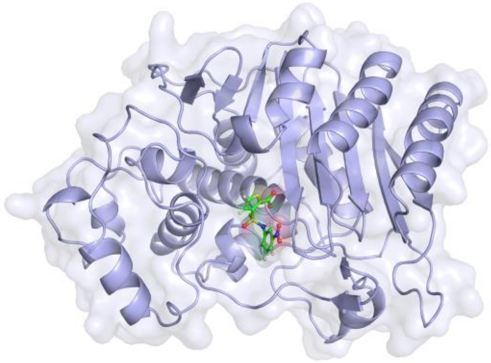 |
| 2 | 1XGJ | 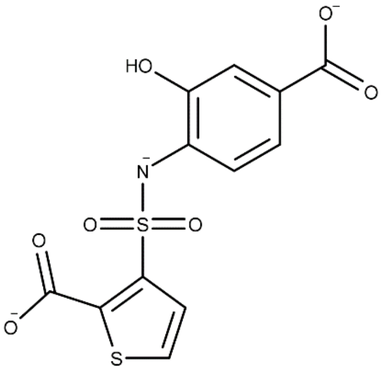 | 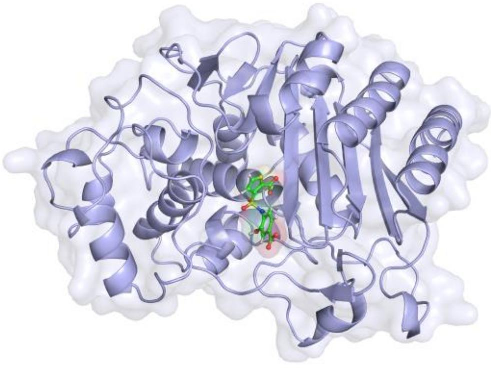 |
| 3 | 2HDU | 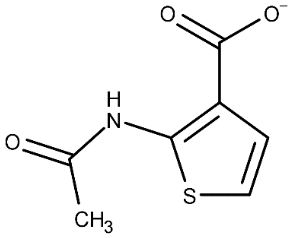 | 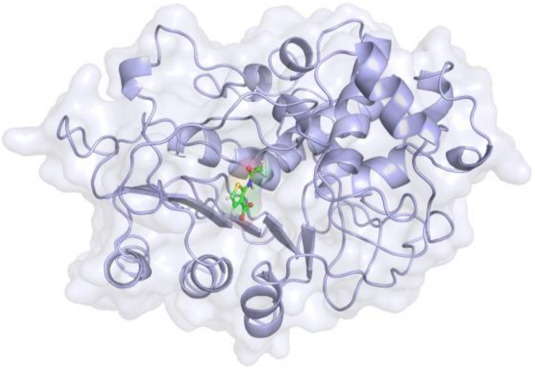 |

| | | | |
|---|---|---|---|
| **4** | **2PU2** | 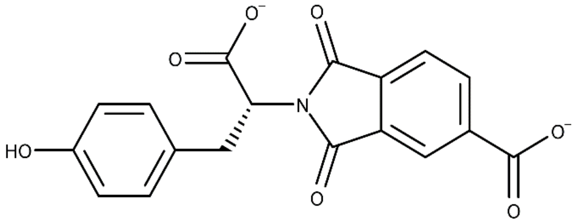 | 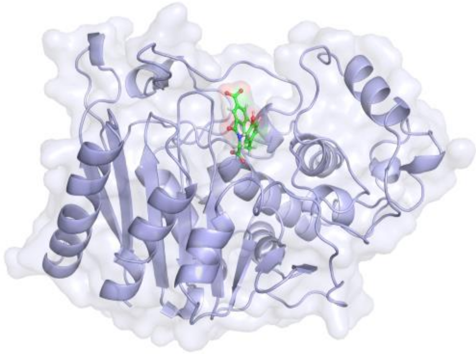 |
| **5** | **2R9W** | 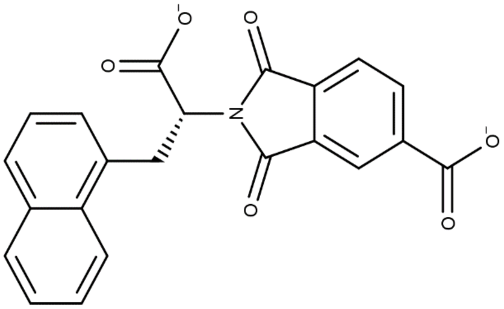 | 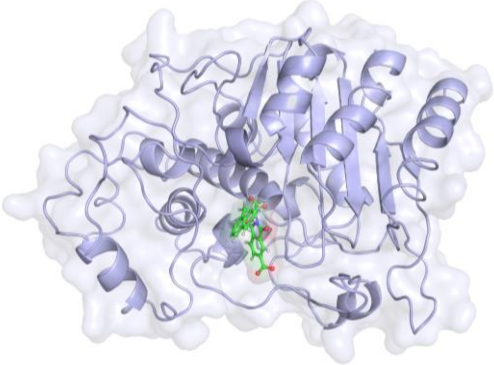 |
| **6** | **2R9X** | 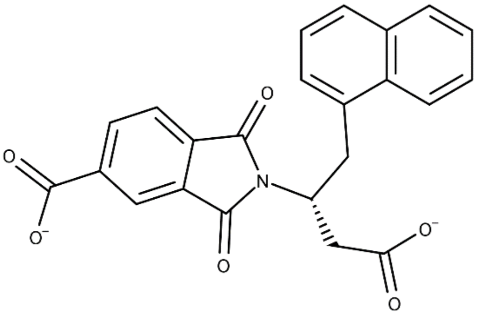 | 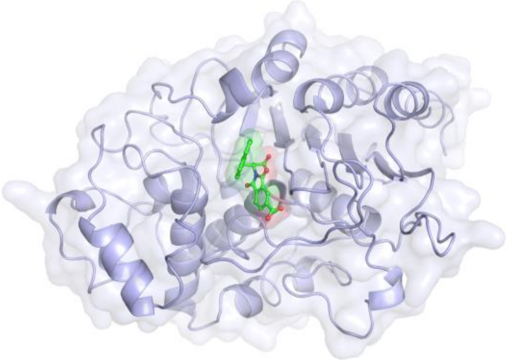 |
| **7** | **3GR2** | 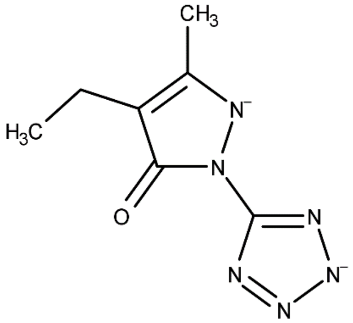 | 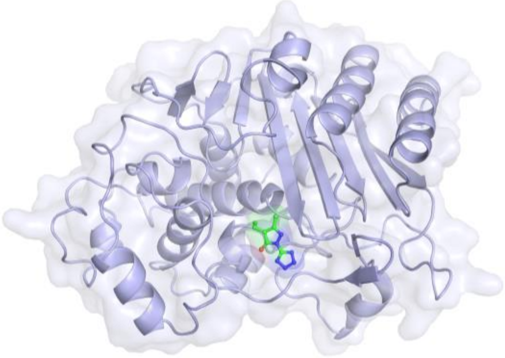 |

| | | | |
|---|---|---|---|
| **8** | **4KZ3** | 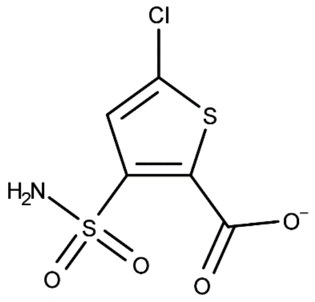 | 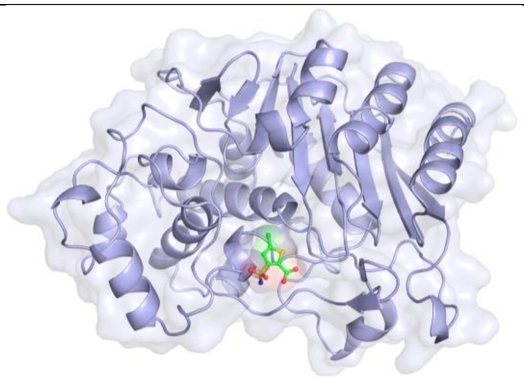 |
| **9** | **4KZ5** | 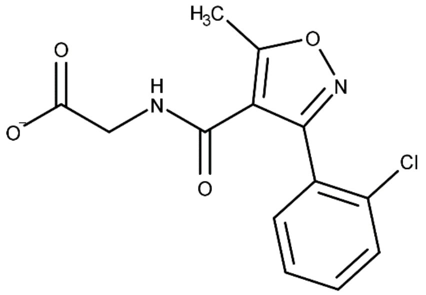 | 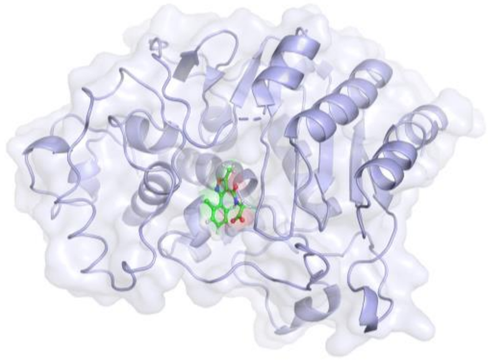 |
| **10** | **4OKP** | 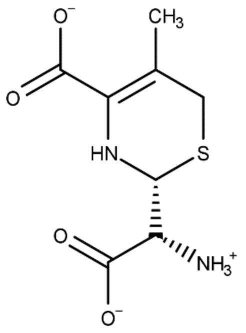 | 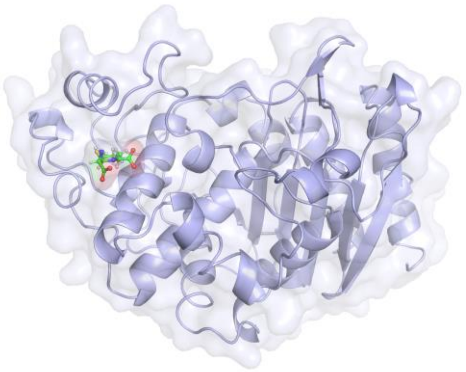 |

| | | | |
|---|---|---|---|
| **11** | **1VSO** | 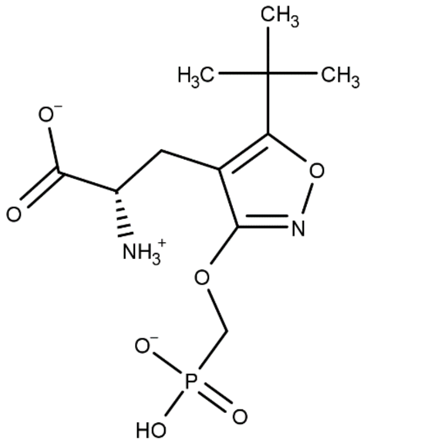 | 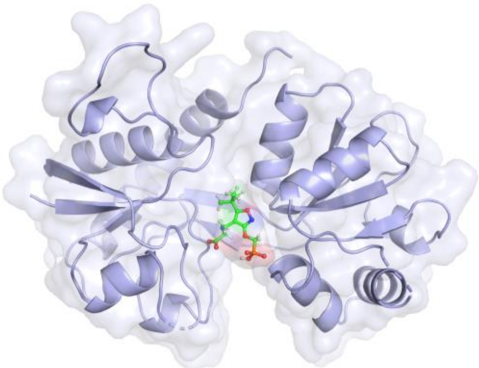 |
| **12** | **2PBW** | 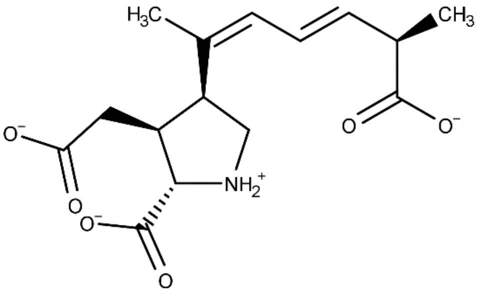 | 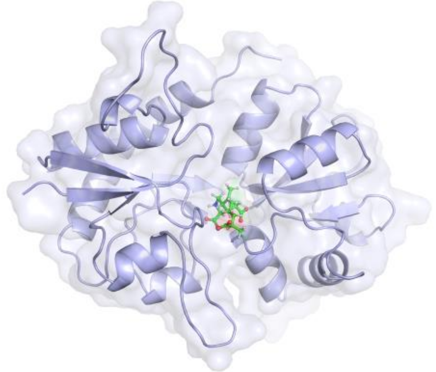 |
| **13** | **2ZNS** | 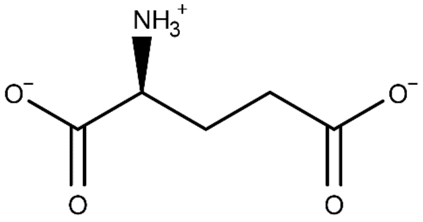 | 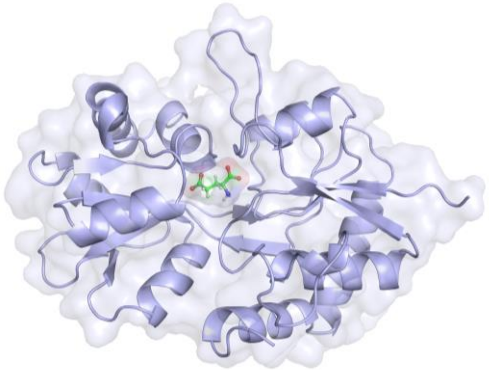 |

| | | | |
|---|---|---|---|
| **14** | **2ZNU** | 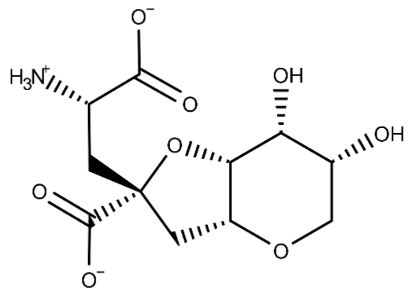 | 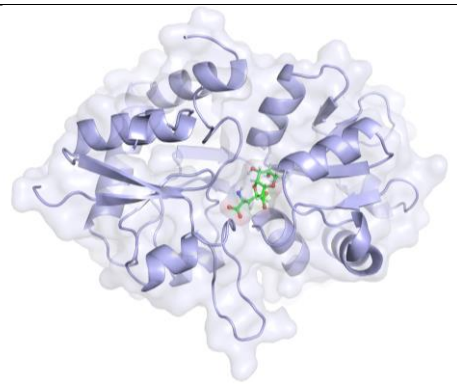 |
| **15** | **3FV1** | 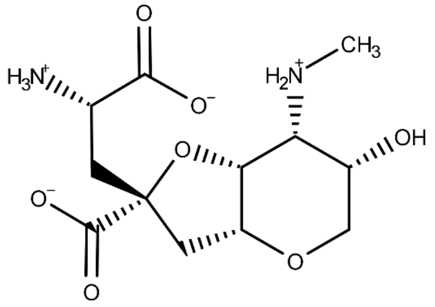 | 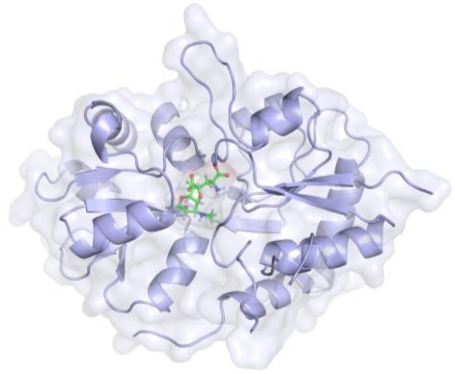 |
| **16** | **3FVN** | 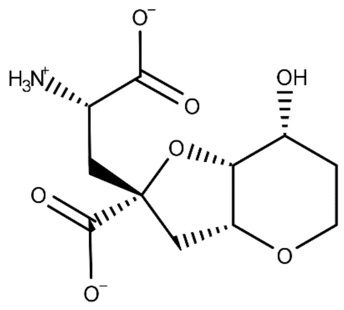 | 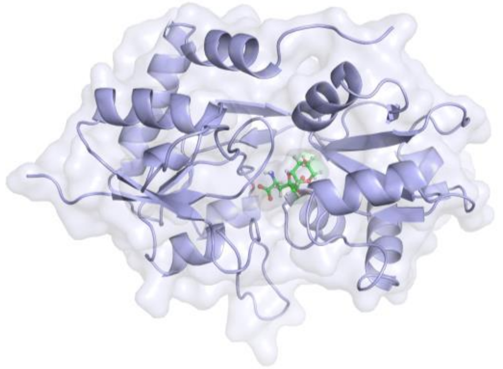 |
| **17** | **3FVG** | 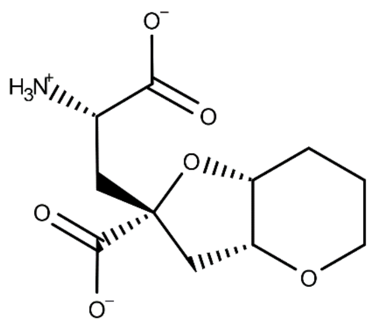 | 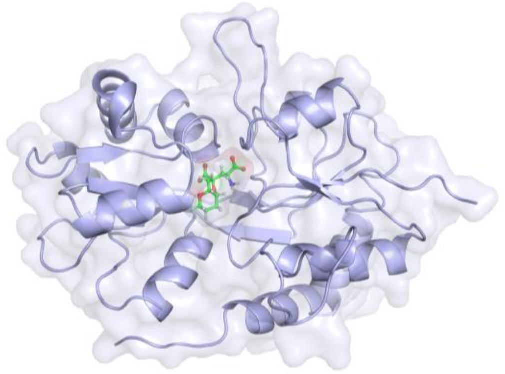 |

| | | | |
|---|---|---|---|
| **18** | **3FVK** | 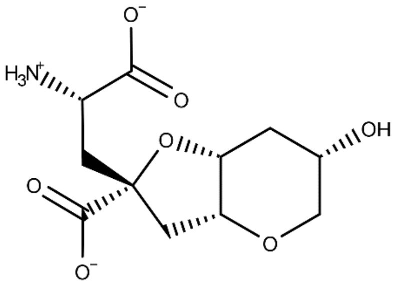 | 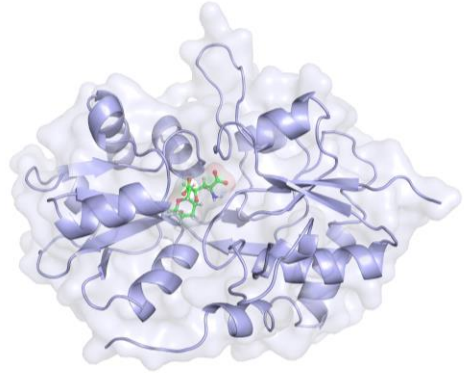 |
| **19** | **4DLD** | 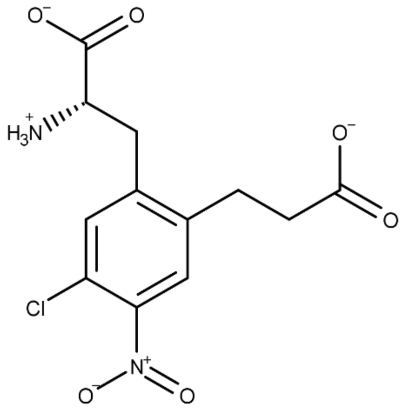 | 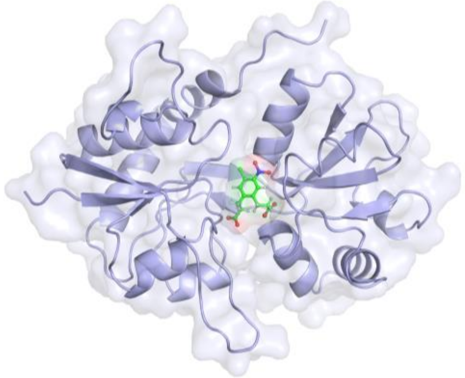 |
| **20** | **4E0X** | 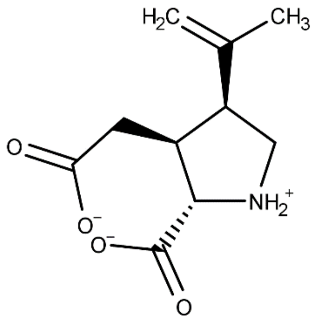 | 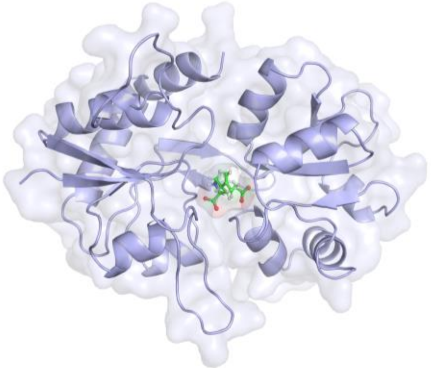 |

| | | | |
|---|---|---|---|
| **21** | **2QG0** | 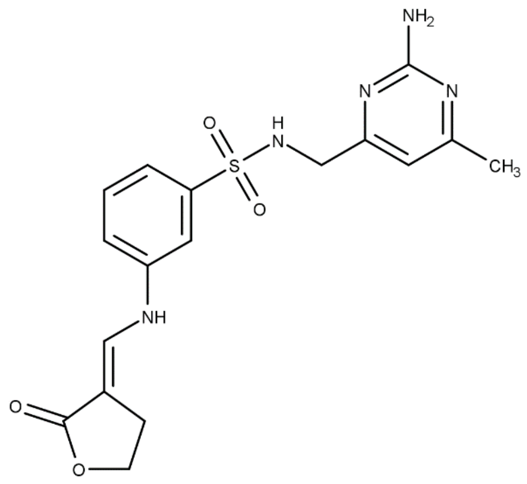 | 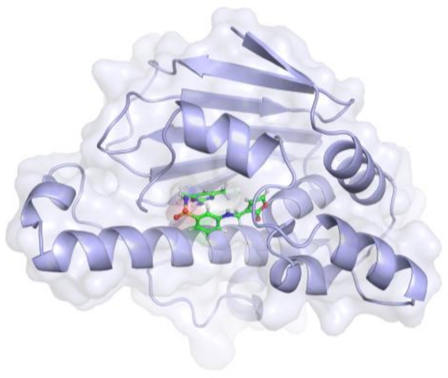 |
| **22** | **2QG2** | 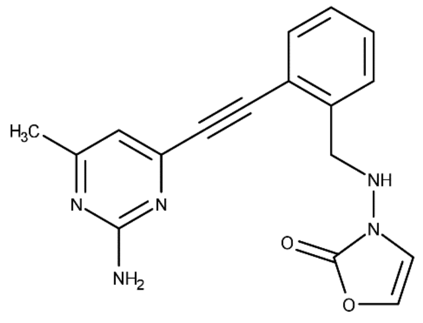 | 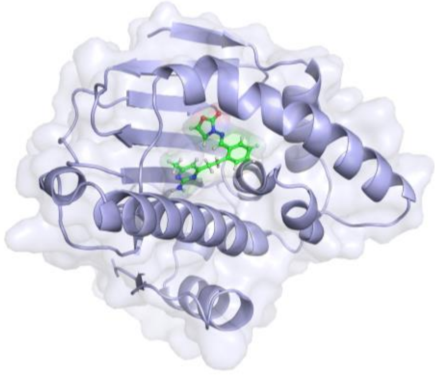 |
| **23** | **3K97** | 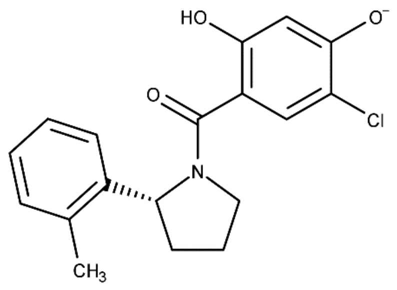 | 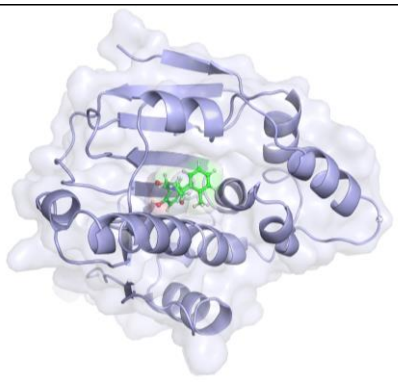 |

| | | | |
|---|---|---|---|
| **24** | **3NMQ** | 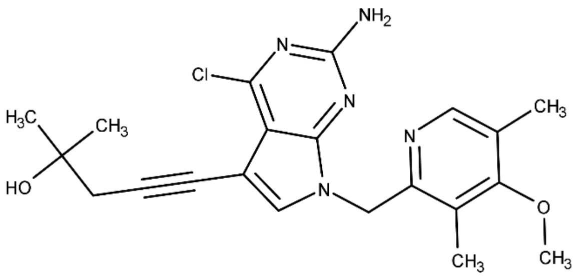 | 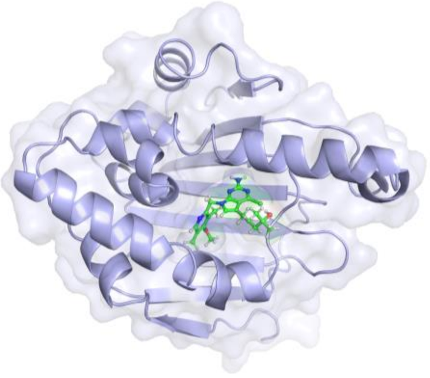 |
| **25** | **3QDD** | 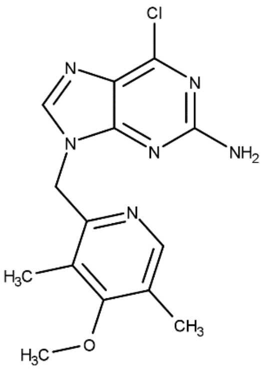 | 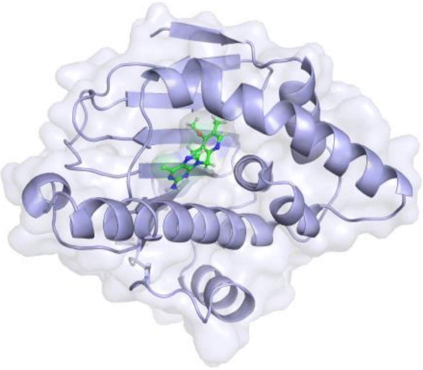 |
| **26** | **3R4M** | 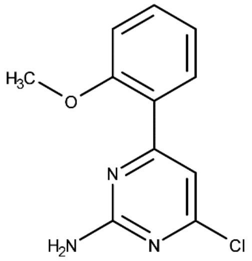 | 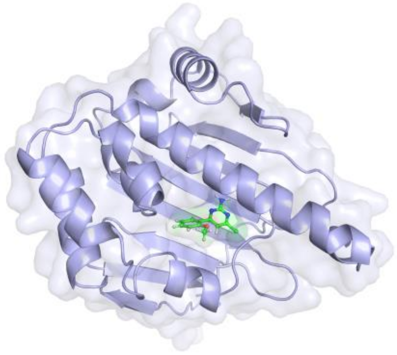 |
| **27** | **4CWF** | 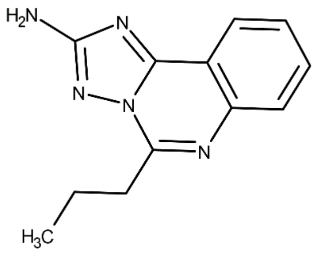 | 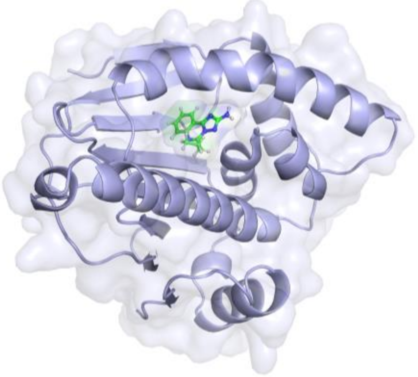 |

| | | | |
|---|---|---|---|
| **28** | **4CWT** | 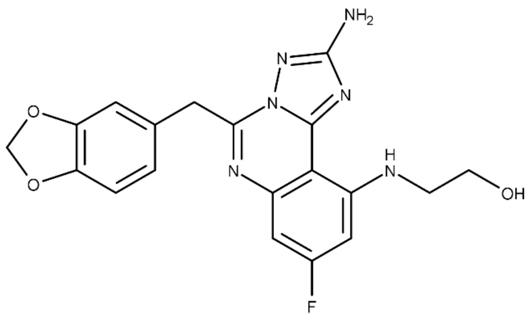 | 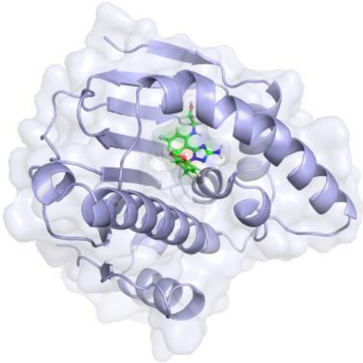 |
| **29** | **4NH8** | 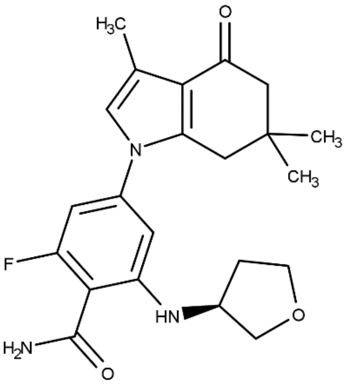 | 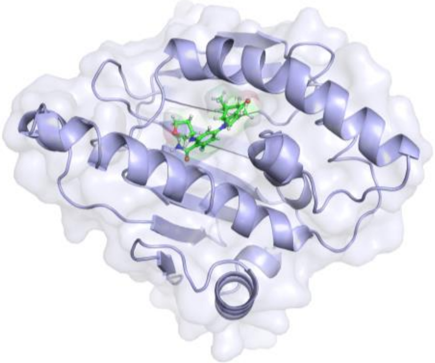 |
| **30** | **4O07** | 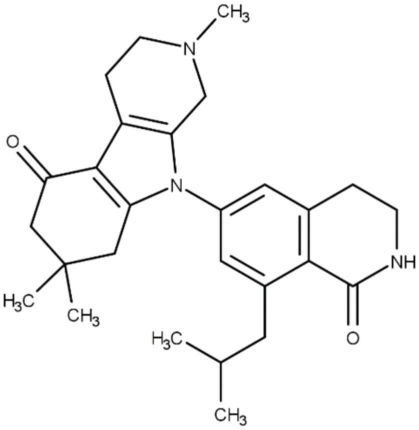 | 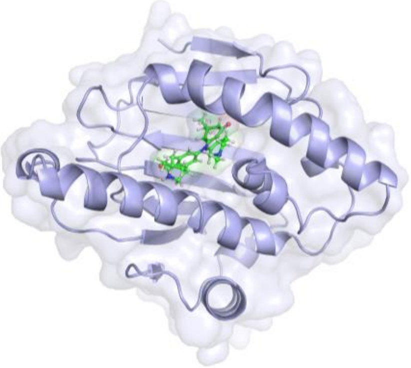 |

| | | | |
|---|---|---|---|
| **31** | **6M2N** | 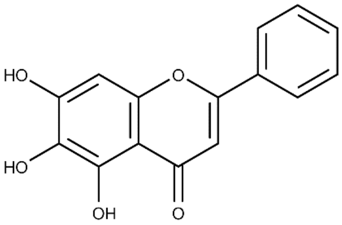 | 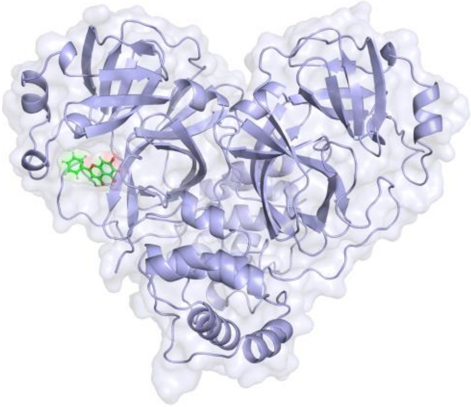 |
| **32** | **6WTT** | 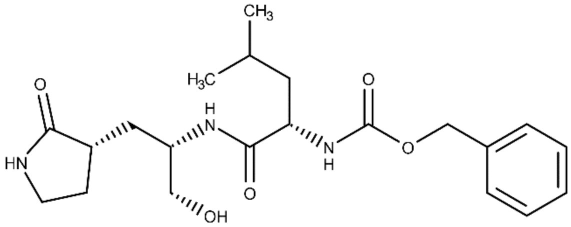 | 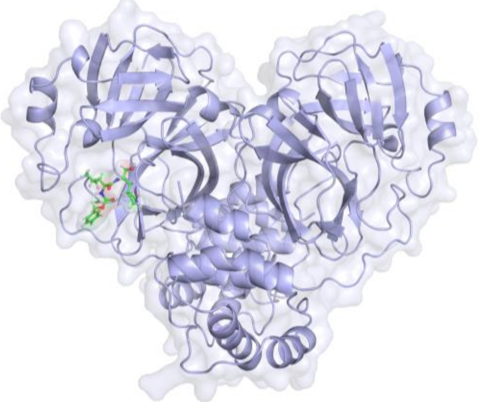 |
| **33** | **6XBG** | 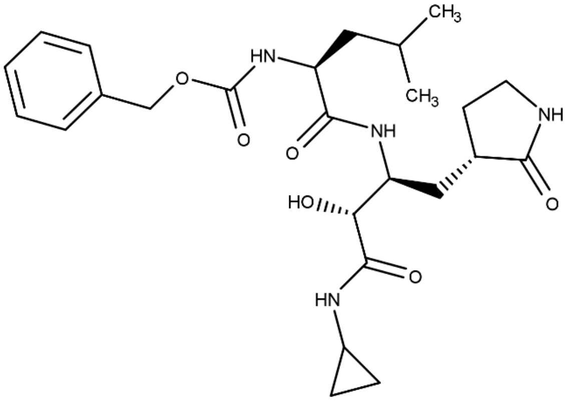 | 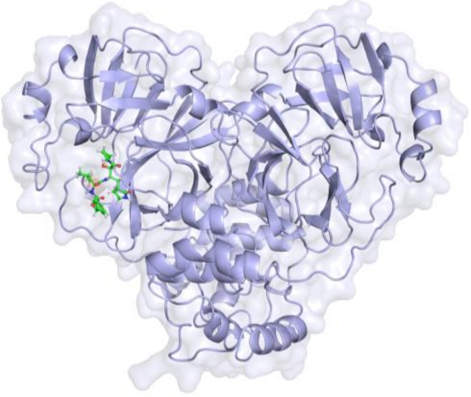 |

| | | | |
|---|---|---|---|
| **34** | **6XMK** | 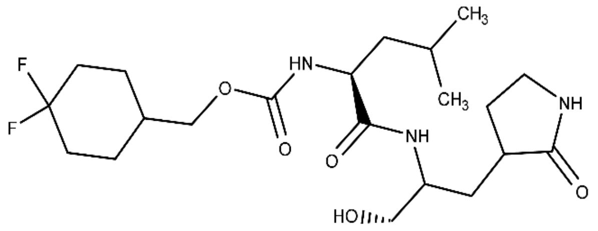 | 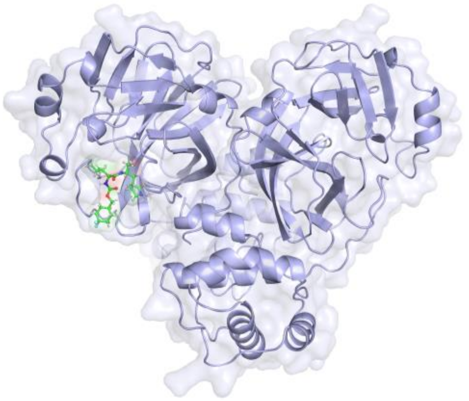 |
| **35** | **7B3E** | 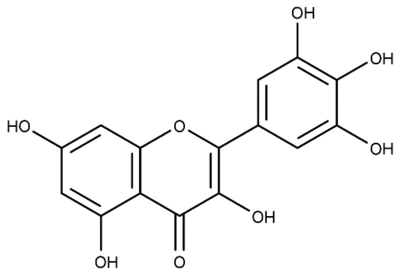 | 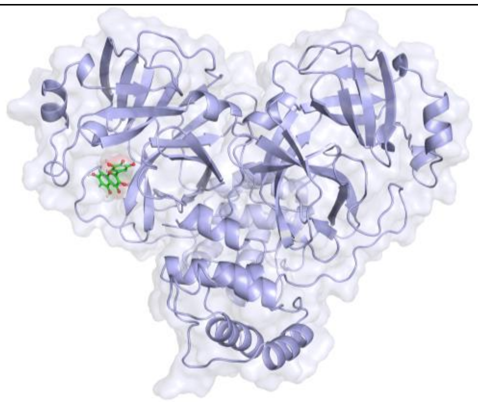 |
| **36** | **7LCR** | 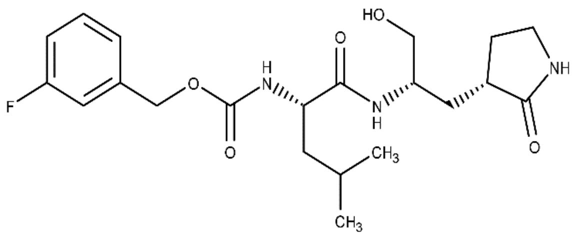 | 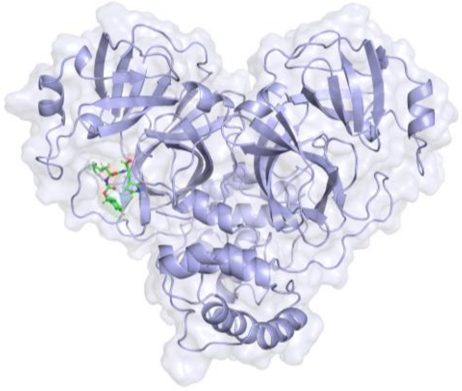 |

| | | | |
|---|---|---|---|
| **37** | **7L8I** | 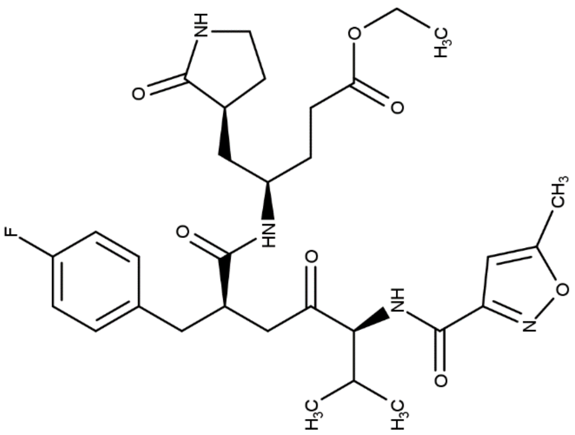 | 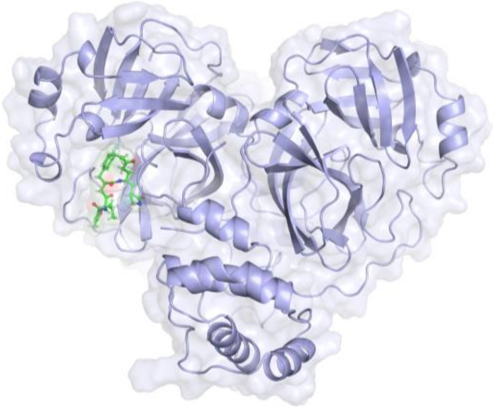 |
| **38** | **7LDL** | 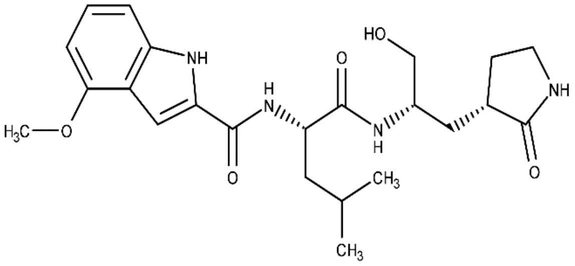 | 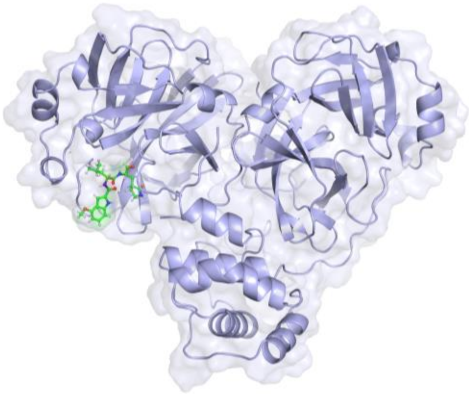 |
| **39** | **7NG3** | 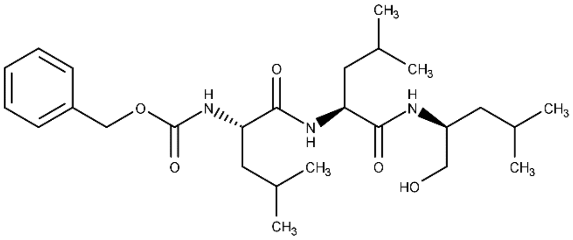 | 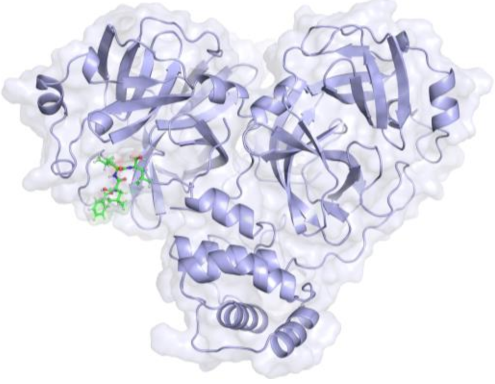 |

| | | | |
|---|---|---|---|
| **40** | **8A4T** | 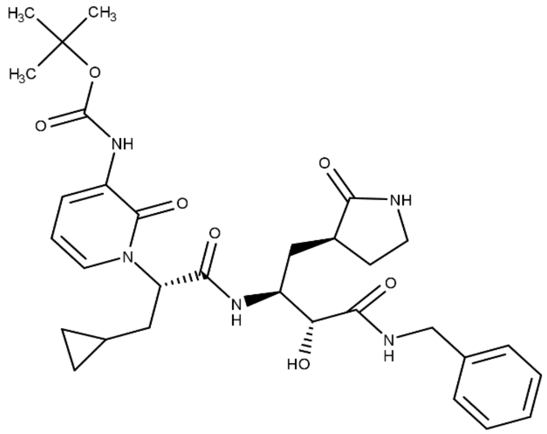 | 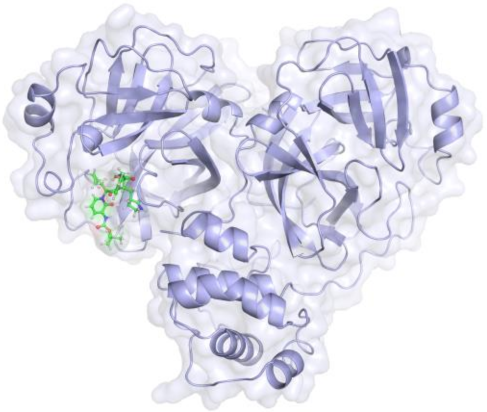 |

**Table S2.** All-atom RMSD of AmpC + ligand and ligand in solution systems.

| $N^0$ | PDB ID | Complex in Solution | Ligand in Solution |
|---|---|---|---|
| 1 | 1XGI | 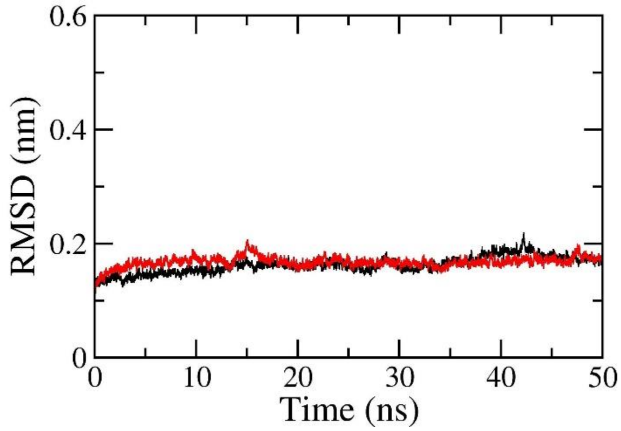 | 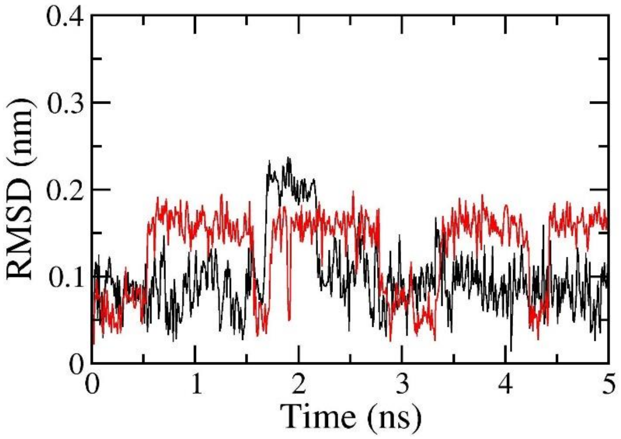 |
| 2 | 1XGJ | 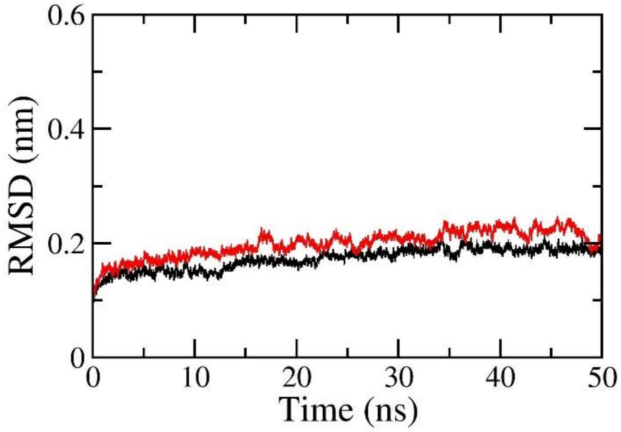 | 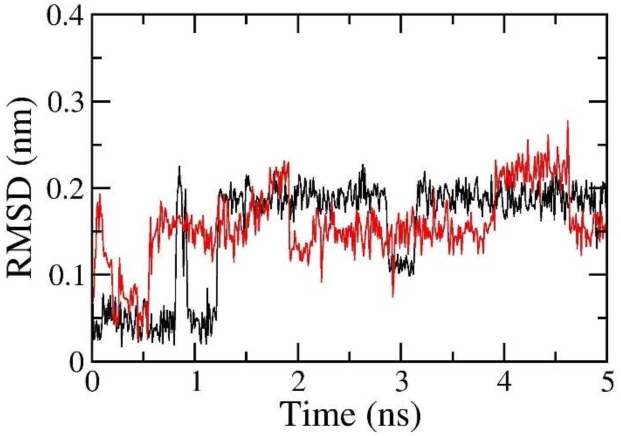 |
| 3 | 2HDU | 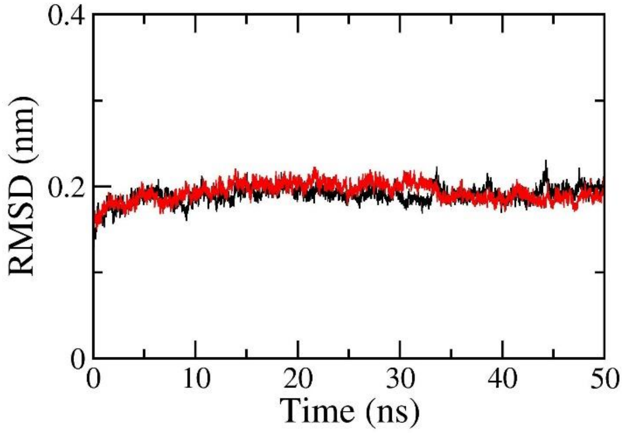 | 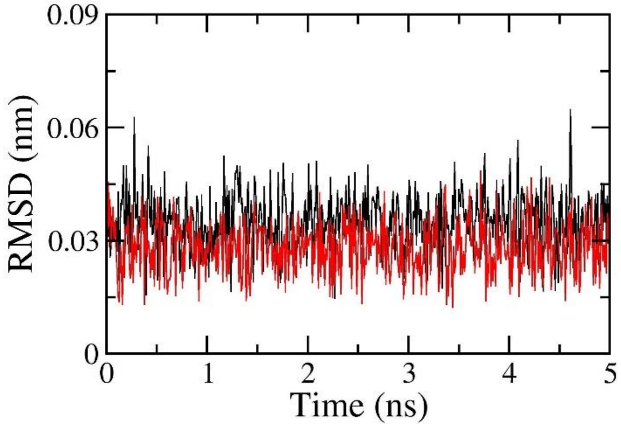 |

<table>
<tr><td>4</td><td>2PU2</td><td>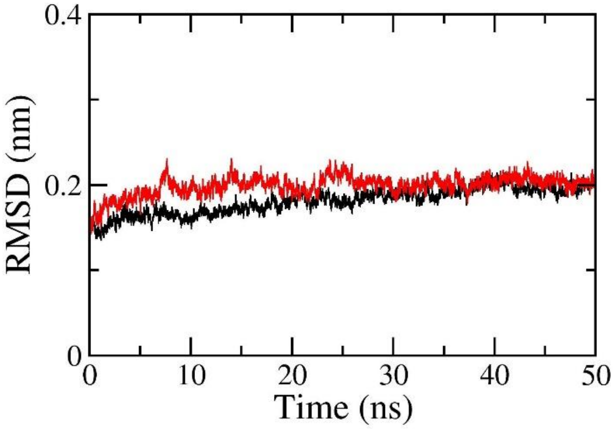
</td><td>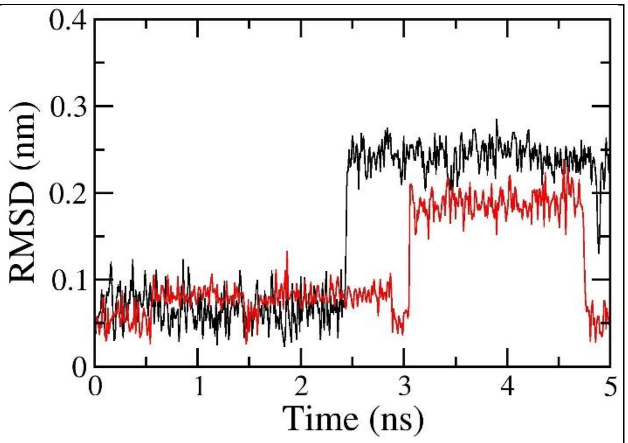
</td></tr>
<tr><td>5</td><td>2R9W</td><td>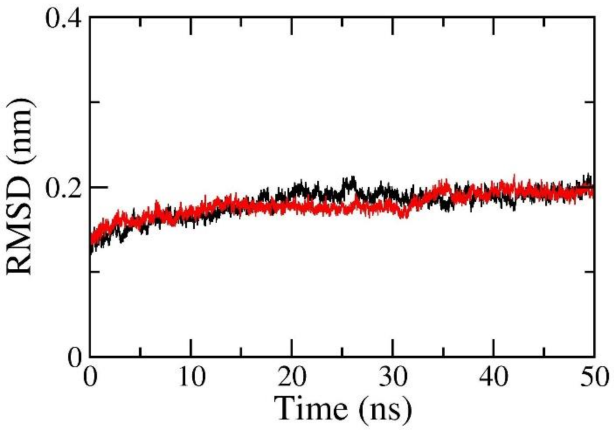
</td><td>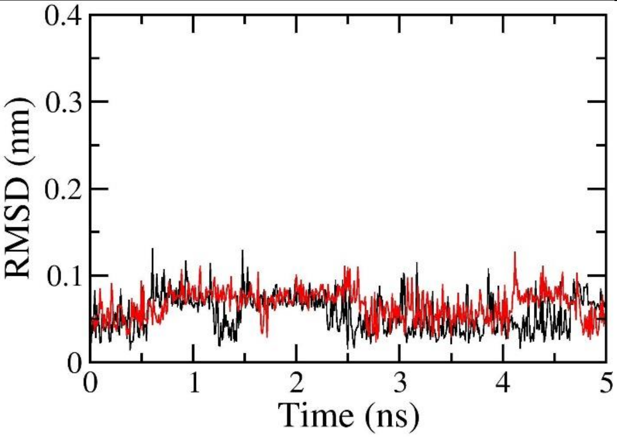
</td></tr>
<tr><td>6</td><td>2R9X</td><td>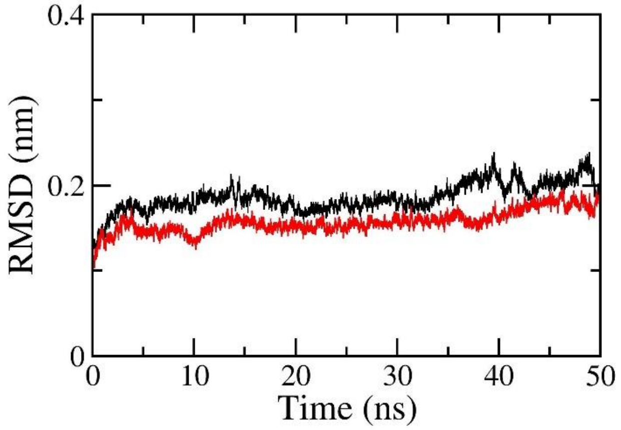
</td><td>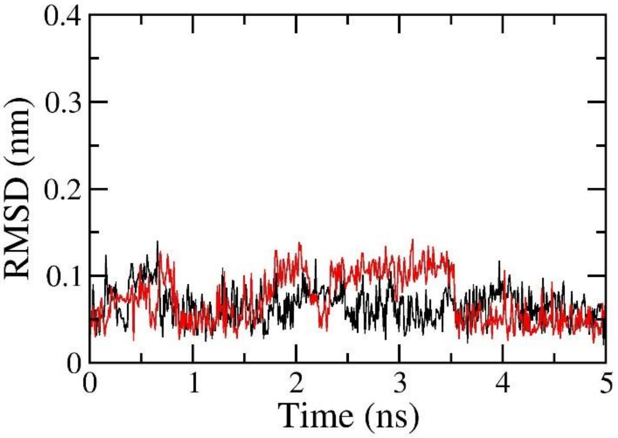
</td></tr>
</table>

<table>
<tr>
<td>7</td>
<td>3GR2</td>
<td>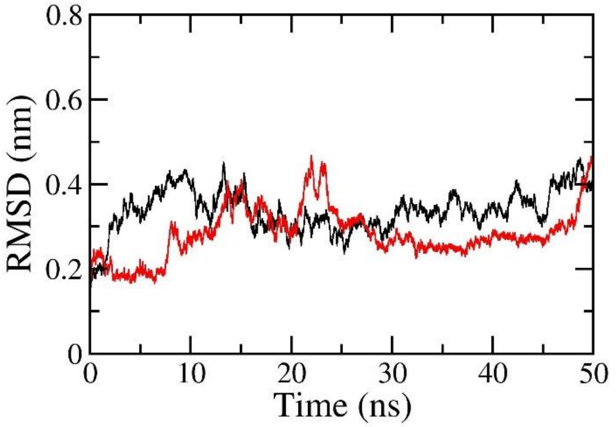
</td>
<td>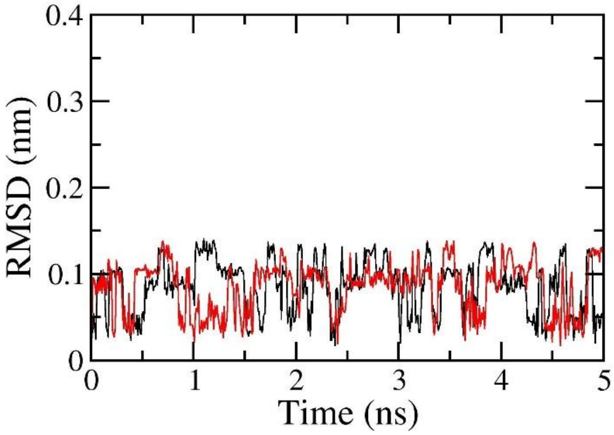
</td>
</tr>
<tr>
<td>8</td>
<td>4KZ3</td>
<td>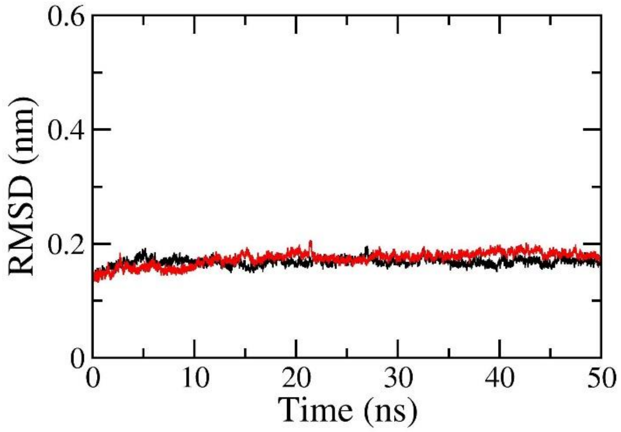
</td>
<td>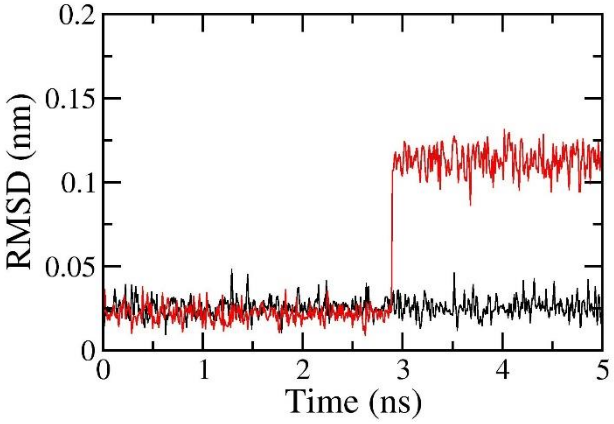
</td>
</tr>
<tr>
<td>9</td>
<td>4KZ5</td>
<td>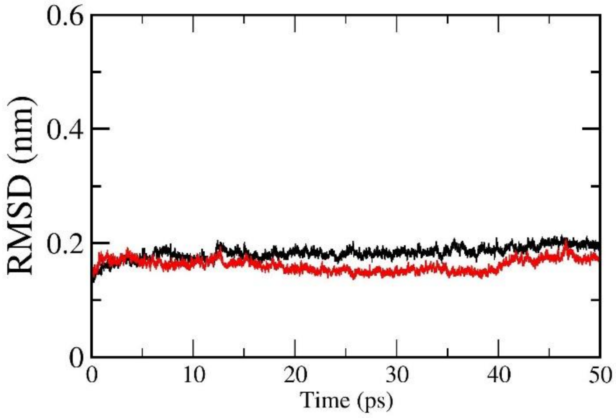
</td>
<td>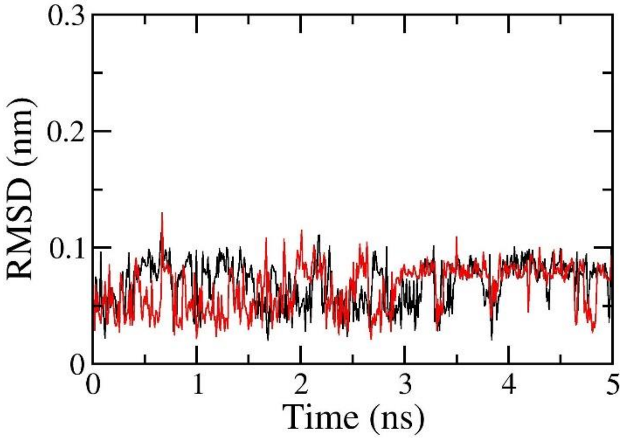
</td>
</tr>
</table>

| | | | |
|---|---|---|---|
| 10 | 4OKP | 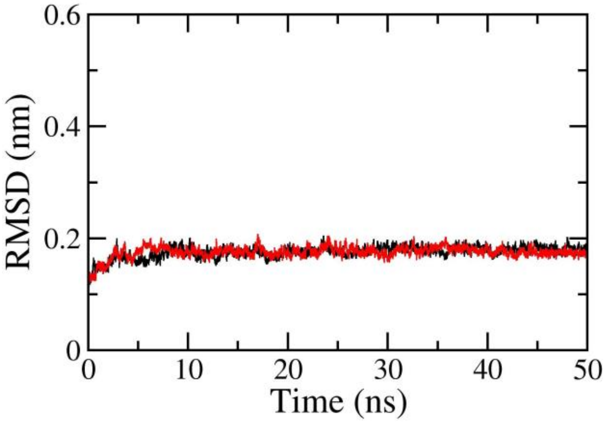RMSD (nm)
Time (ns)
0.6
0.4
0.2
0
0
10
20
30
40
50 | 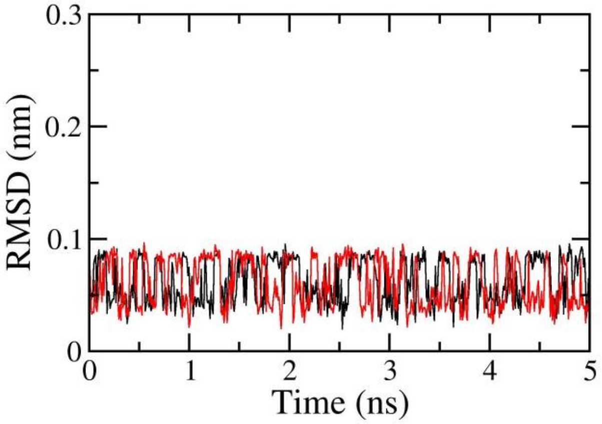RMSD (nm)
Time (ns)
0.3
0.2
0.1
0
0
1
2
3
4
5 |

**Table S3.** All-atom RMSD of GluK1 + ligand and ligand in solution systems.

<table>
<tr><th>N⁰</th><th>PDB ID</th><th>Complex in Solution</th><th>Ligand in Solution</th></tr>
<tr><td>1</td><td>1VSO</td><td>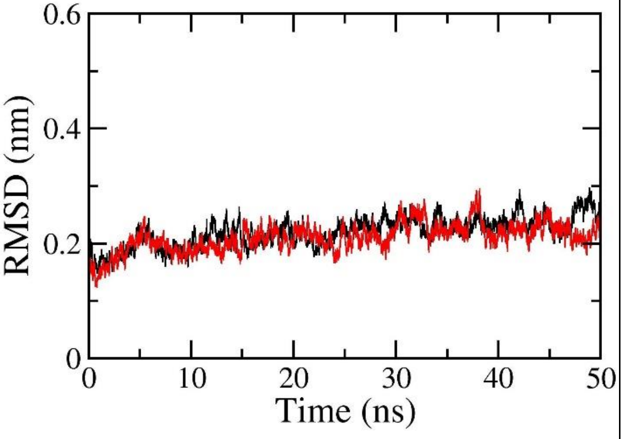
</td><td>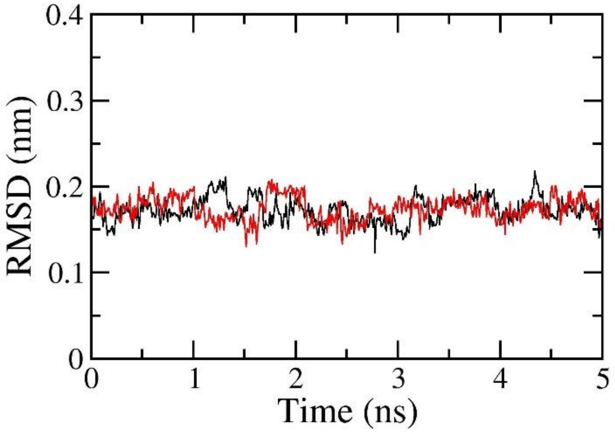
</td></tr>
<tr><td>2</td><td>2PBW</td><td>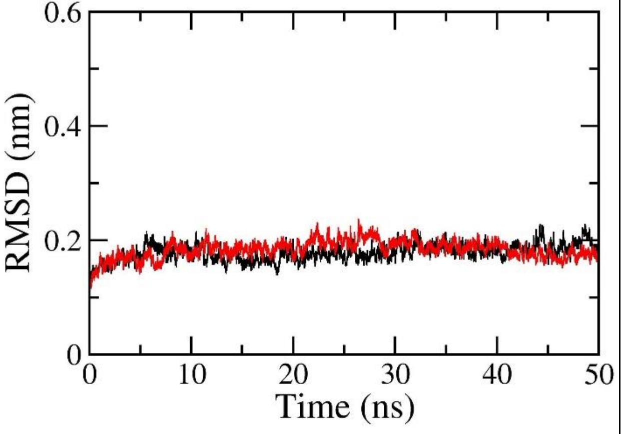
</td><td>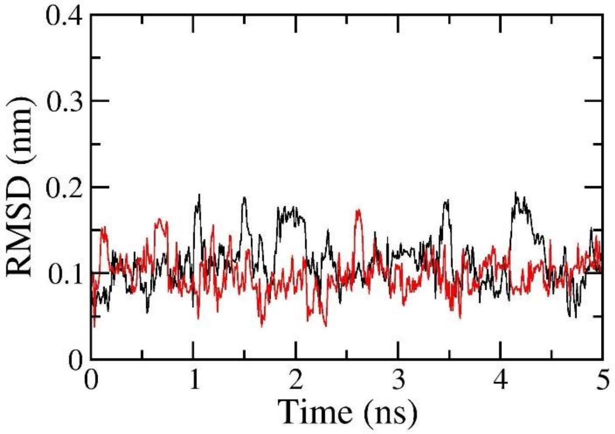
</td></tr>
<tr><td>3</td><td>2ZNS</td><td>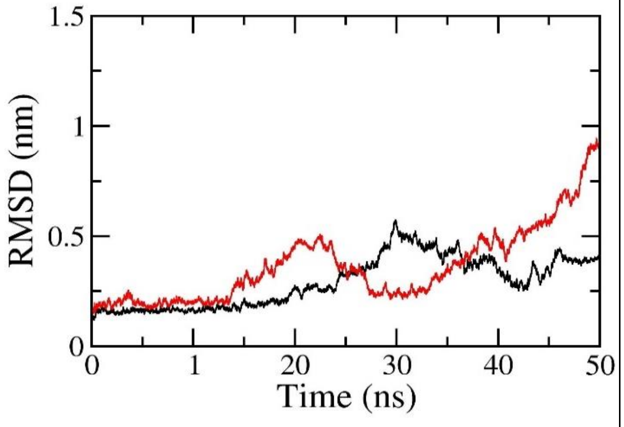
</td><td>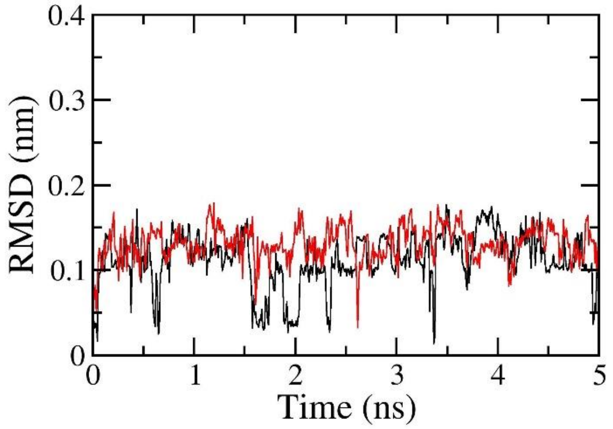
</td></tr>
</table>

<table>
<tr><td>4</td><td>2ZNU</td><td>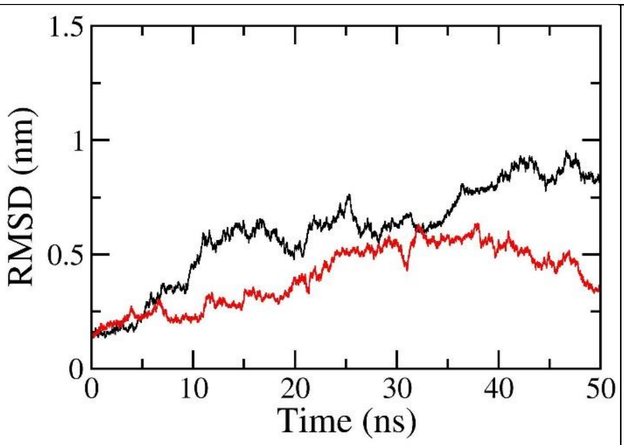</td><td>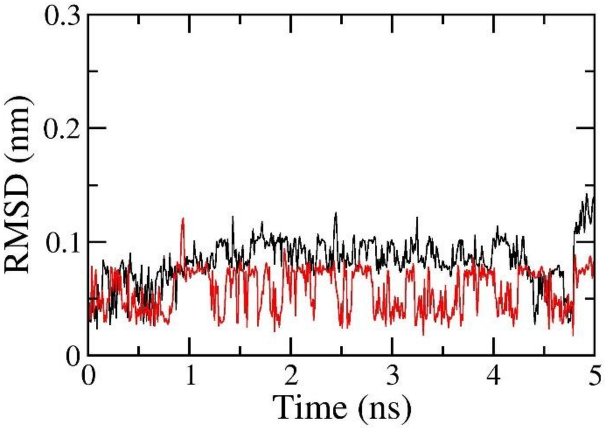</td></tr>
<tr><td>5</td><td>3FKV</td><td>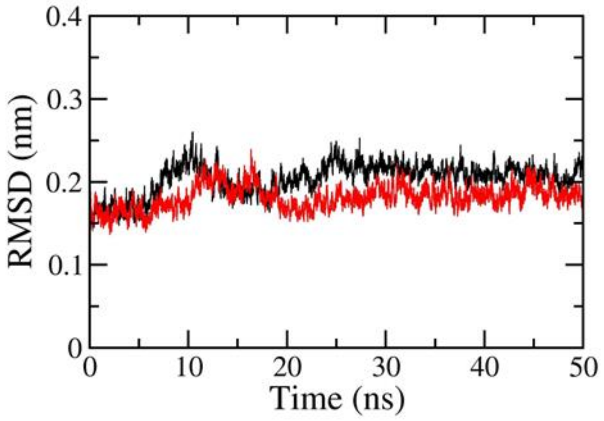</td><td>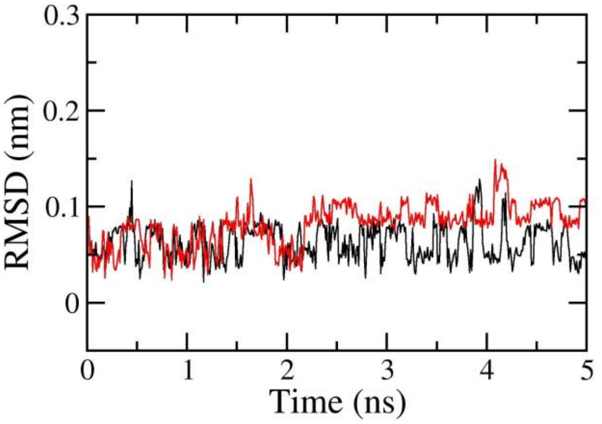</td></tr>
<tr><td>6</td><td>3FVN</td><td></td><td></td></tr>
<tr><td>7</td><td>3FVG</td><td>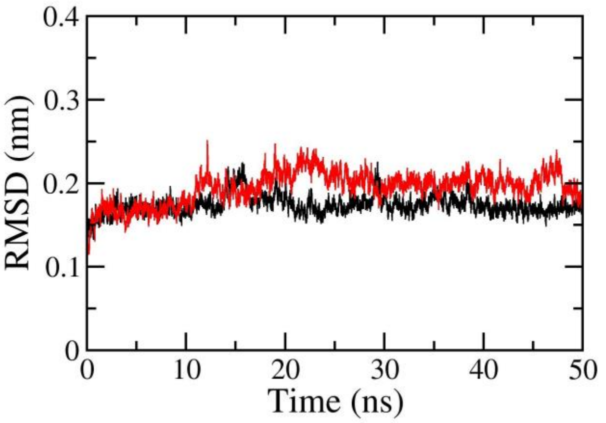</td><td>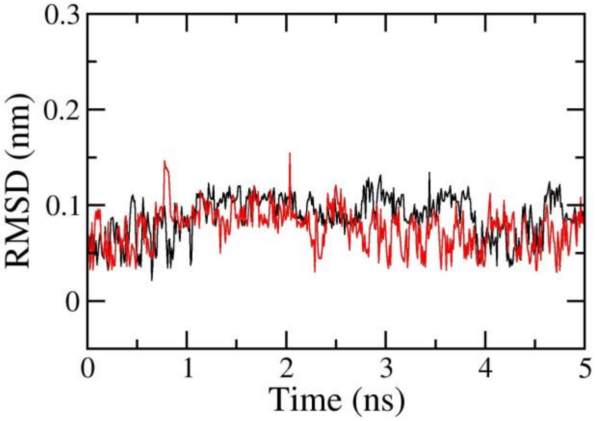</td></tr>
</table>

| | | | |
|---|---|---|---|
| **8** | **3VF1** | 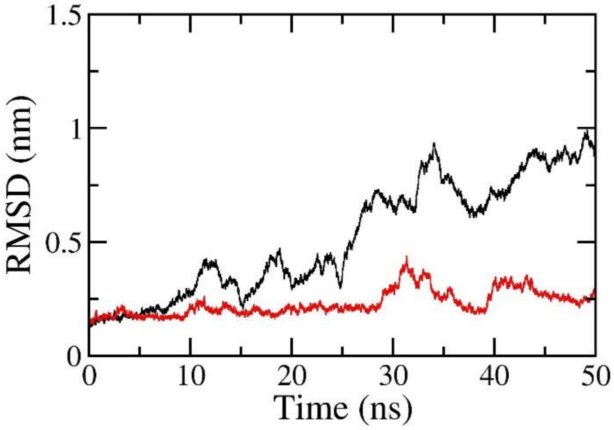 | 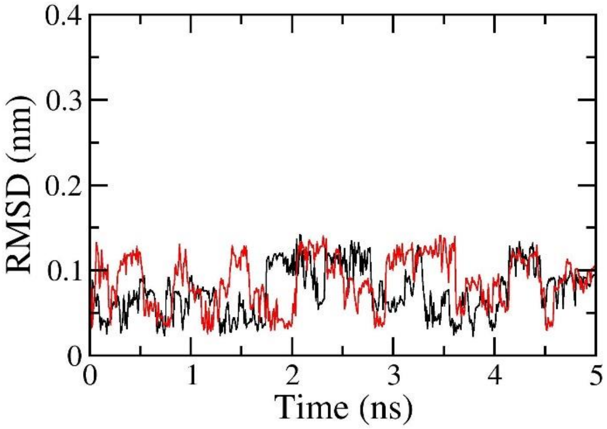 |
| **9** | **4DLD** | 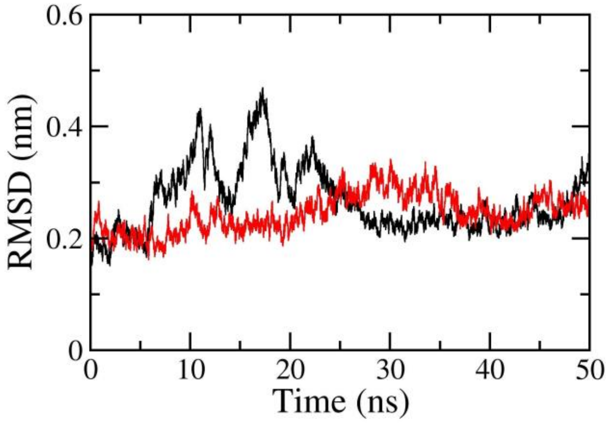 | 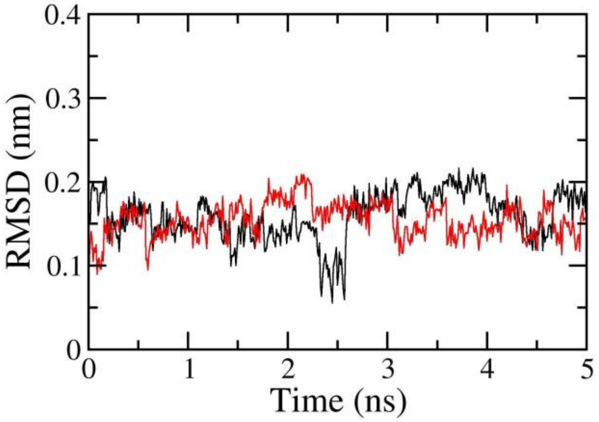 |
| **10** | **4E0X** | 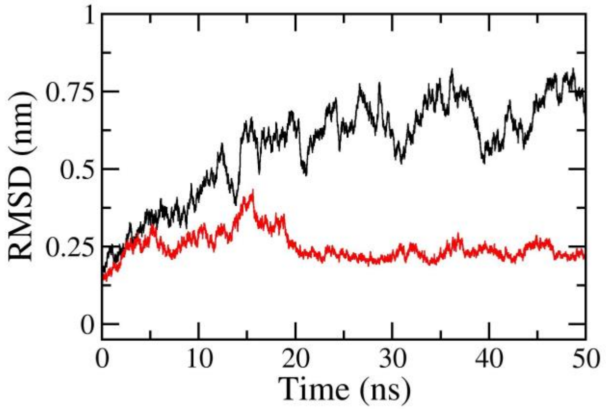 | 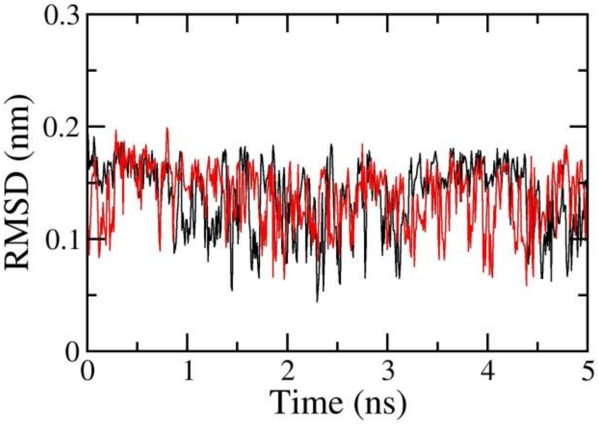 |

**Table S4.** All-atom RMSD of Hsp90 + ligand and ligand in solution systems.

| $N^0$ | PDB ID | Complex in Solution | Ligand in Solution |
|---|---|---|---|
| **1** | **2QG0** | 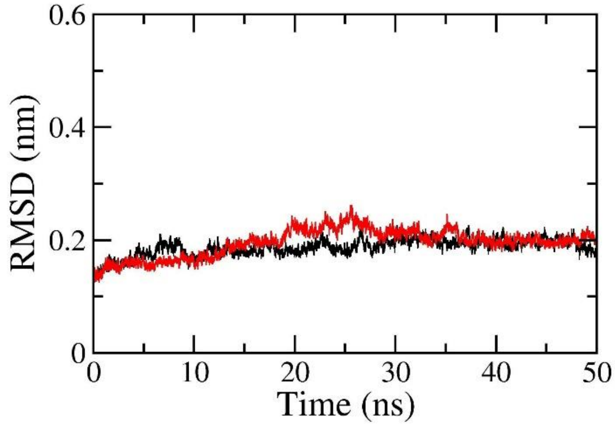  | 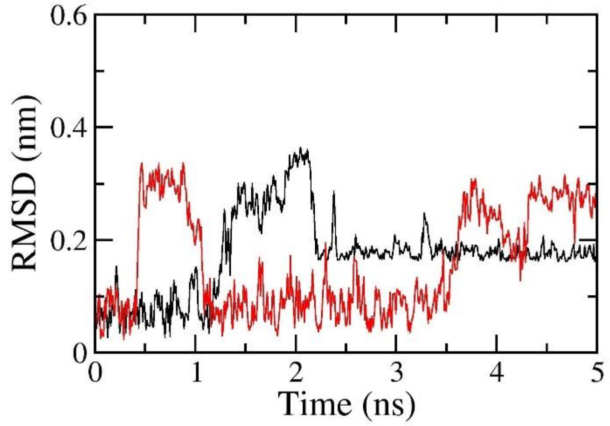  |
| **2** | **2QG2** | 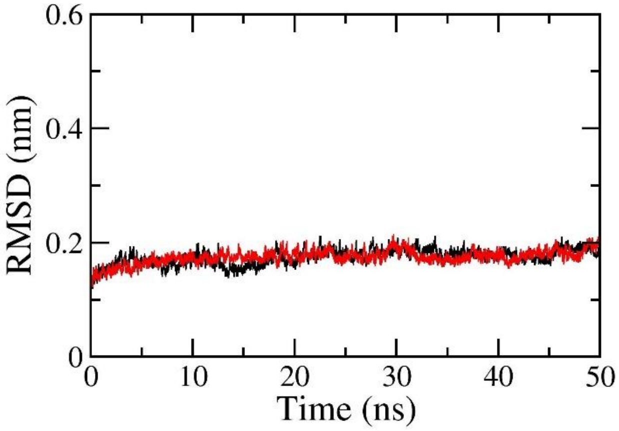  | 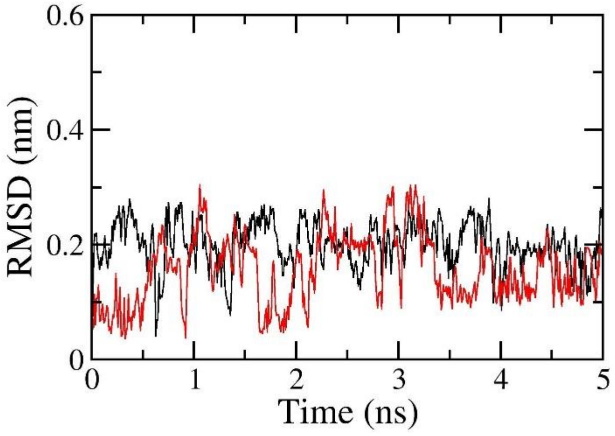  |
| **3** | **3K97** | 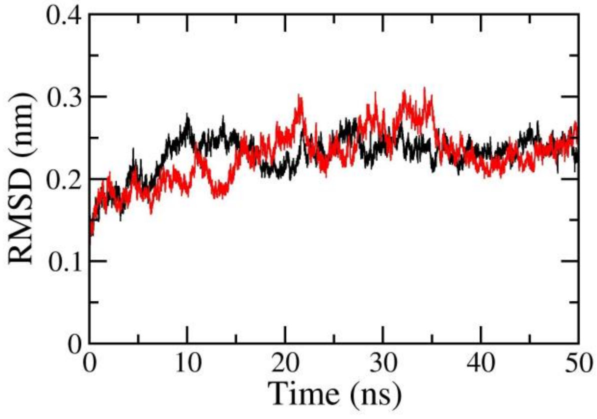  | 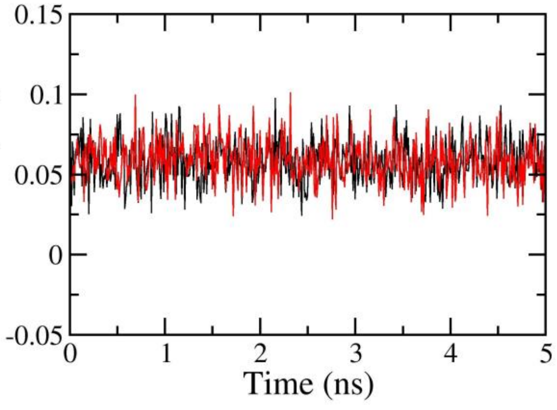  |

| | | | |
|---|---|---|---|
| **4** | **3NMQ** | 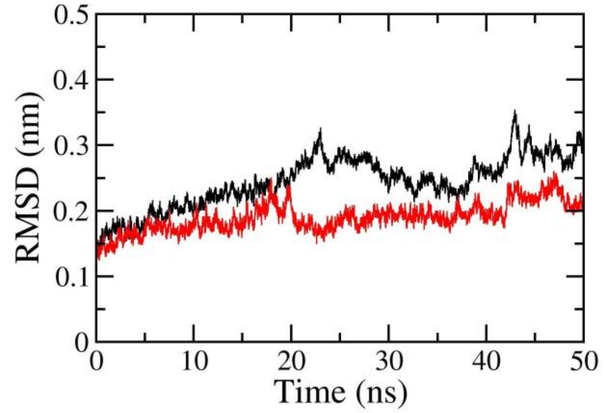 | **S**<br>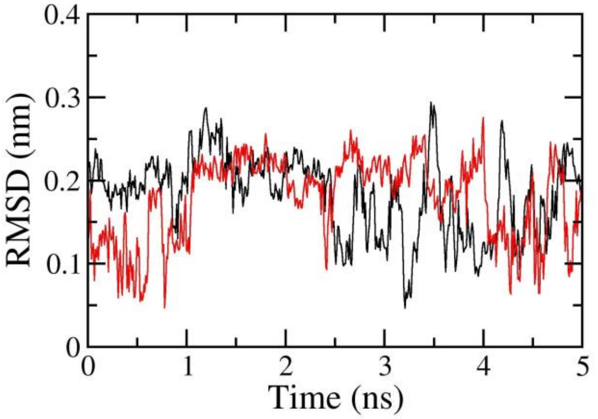 |
| **5** | **3QDD** | 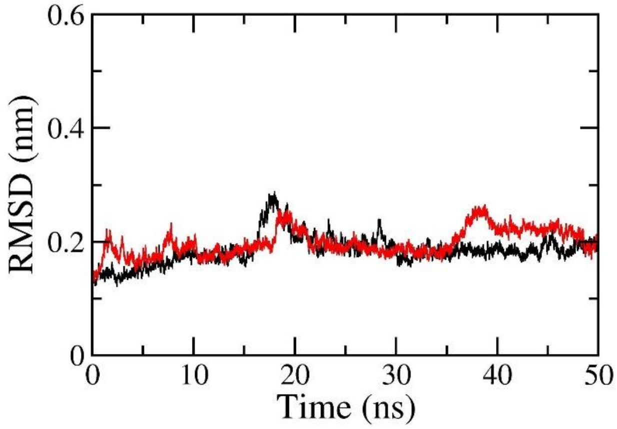 | 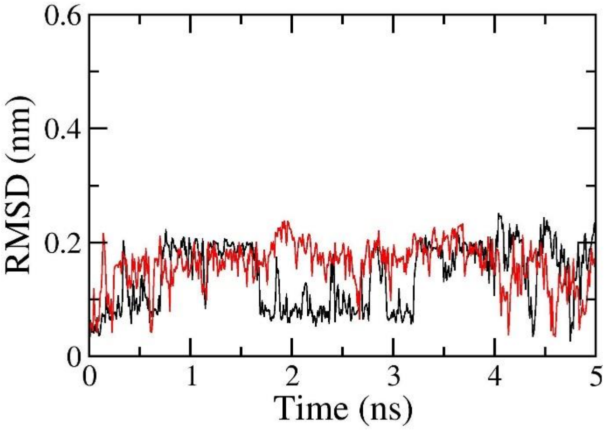 |
| **6** | **3R4M** | 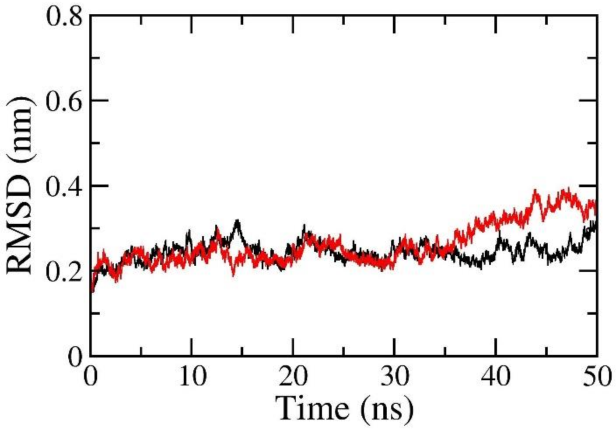 | 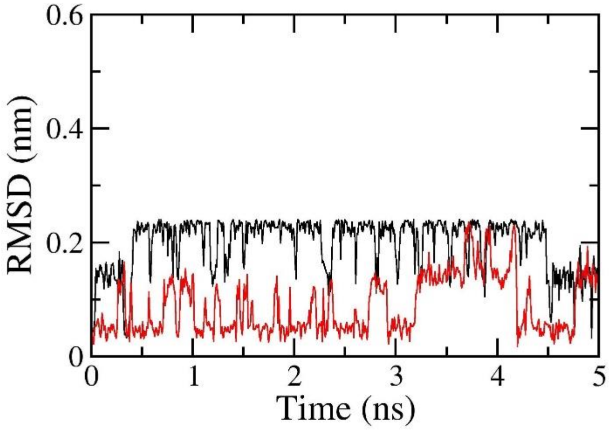 |

| | | | |
|---|---|---|---|
| **7** | **4CWF** | 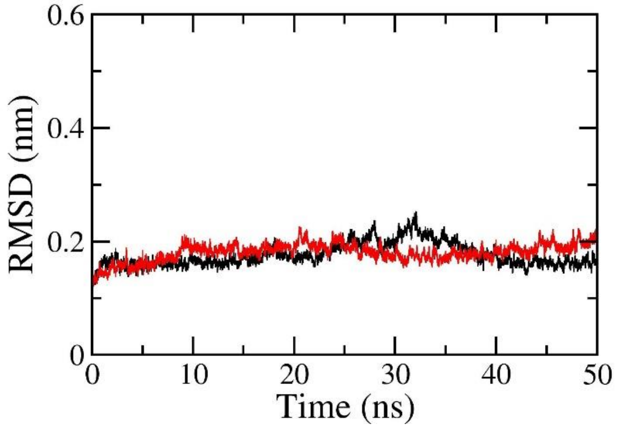  | 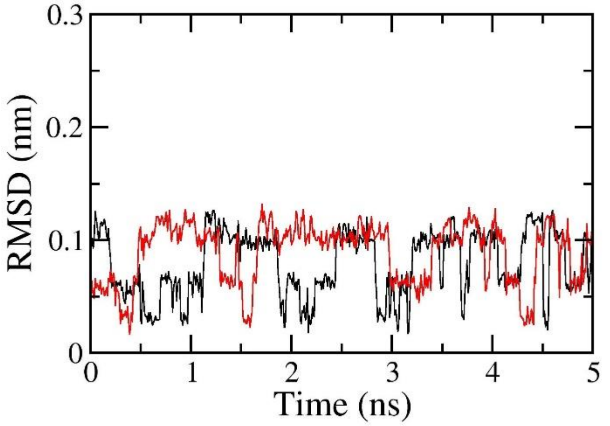  |
| **8** | **4CWT** | 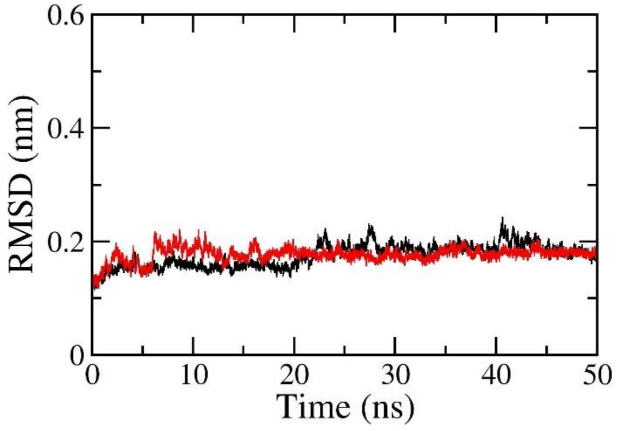  | 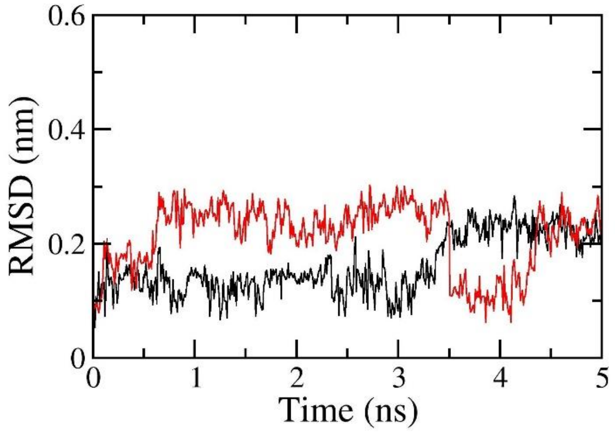  |
| **9** | **4NH8** | 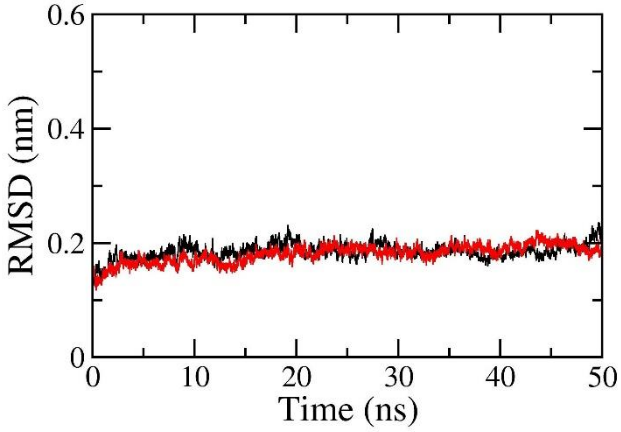  | 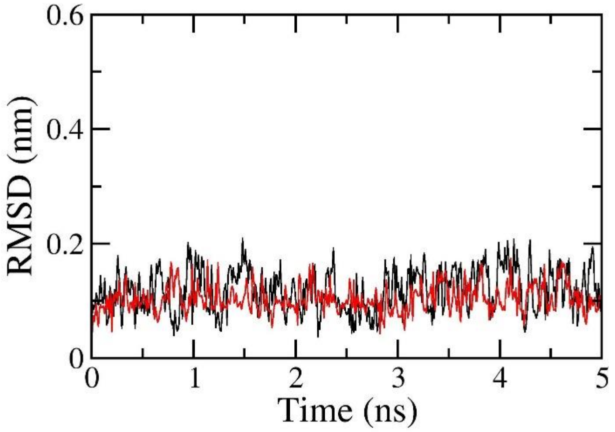  |

| | | | |
|---|---|---|---|
| **10** | **4O07** | 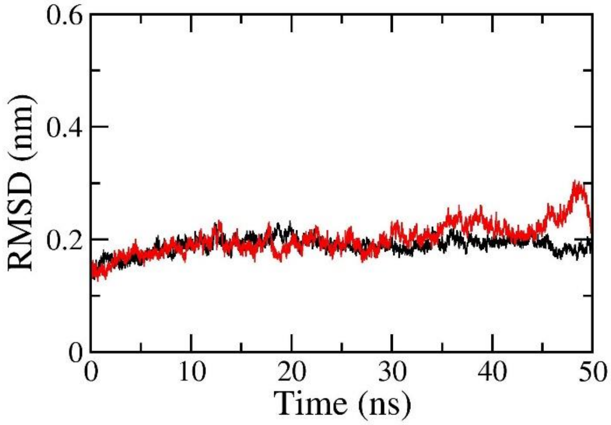0.6<br>0.4<br>0.2<br>0<br>RMSD (nm)<br>0<br>10<br>20<br>30<br>40<br>50<br>Time (ns) | 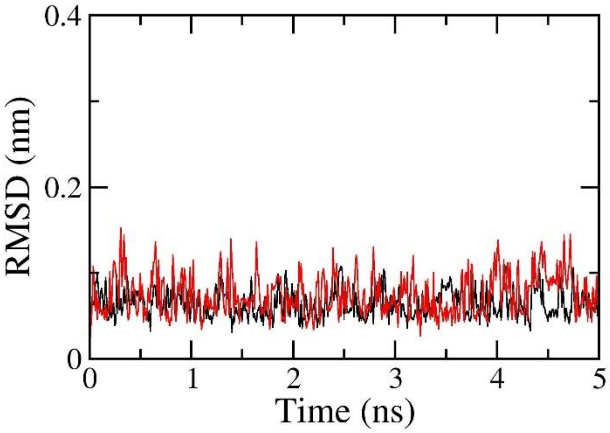0.4<br>0.2<br>0<br>RMSD (nm)<br>0<br>1<br>2<br>3<br>4<br>5<br>Time (ns) |

**Table S5.** All-atom RMSD of SARS-CoV-2 Mpro + ligand and ligand in solution systems.

| N⁰ | PDB ID | Complex in Solution | Ligand in Solution |
|---|---|---|---|
| 1 | 6M2N | 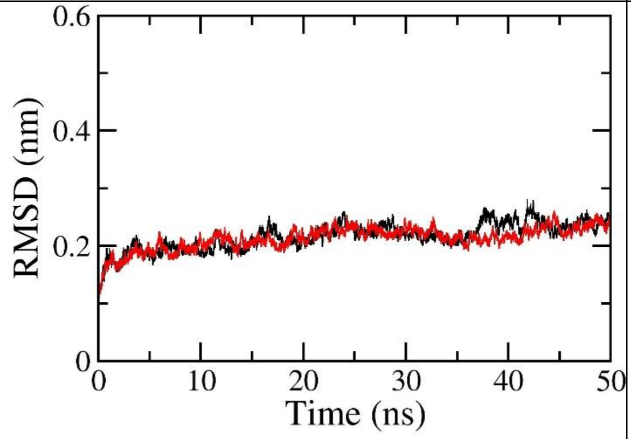  | 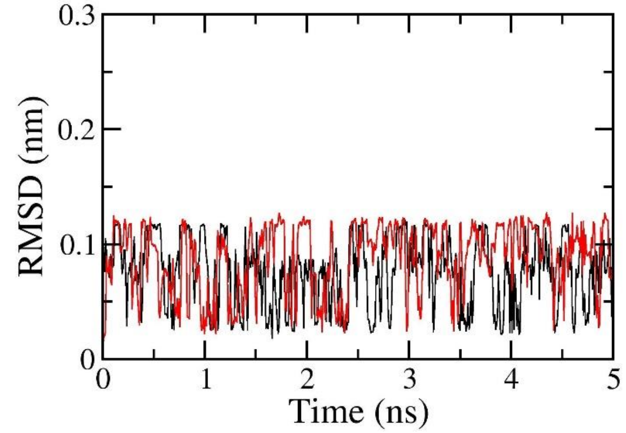  |
| 2 | 6WTT | 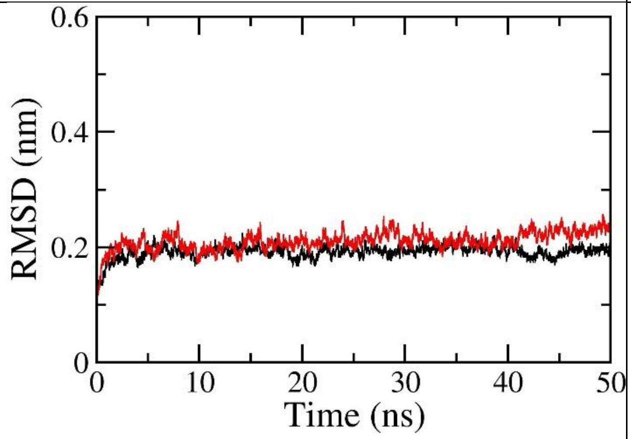  | 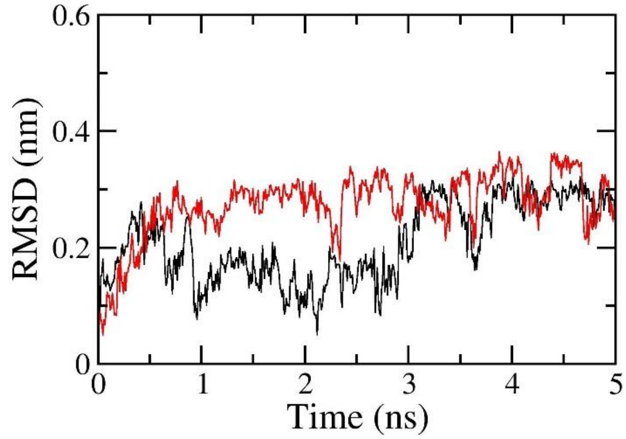  |
| 3 | 6XBG | 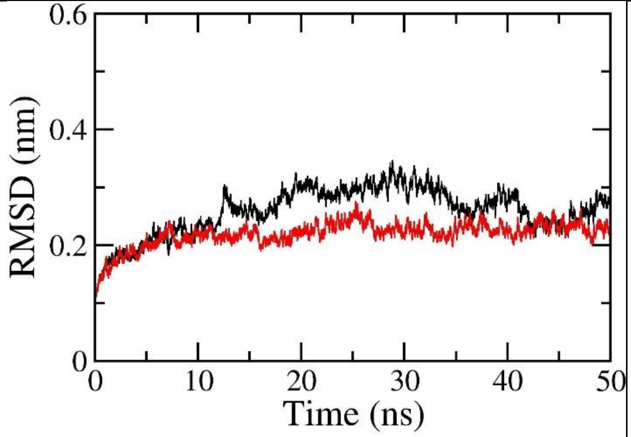  | 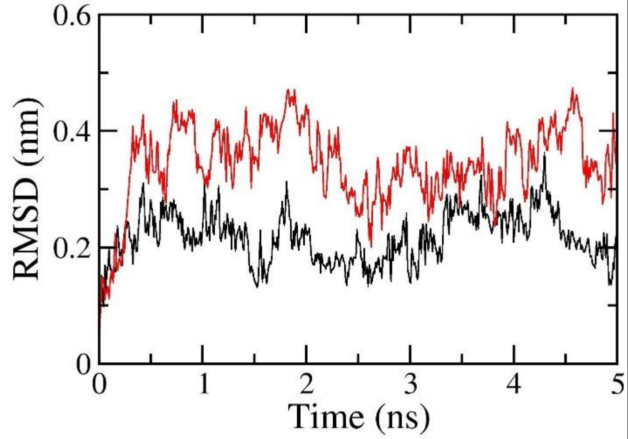  |

| | | | |
|---|---|---|---|
| 4 | 6XMK | 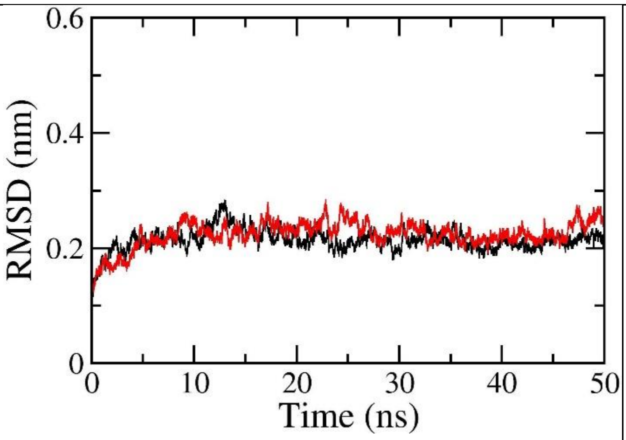 | 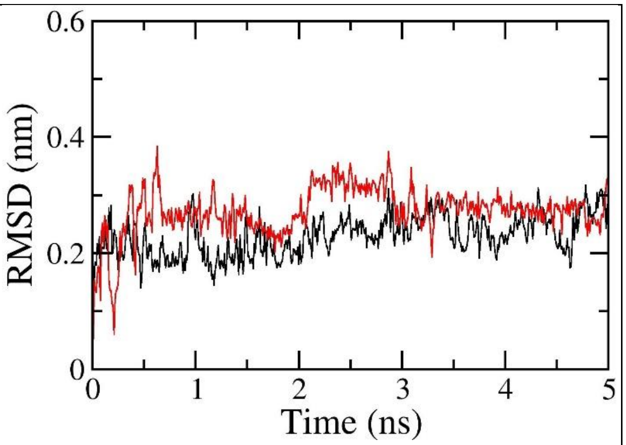 |
| 5 | 7B3E | 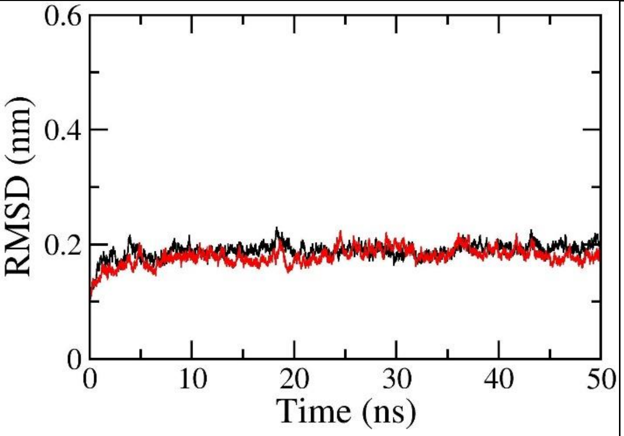 | 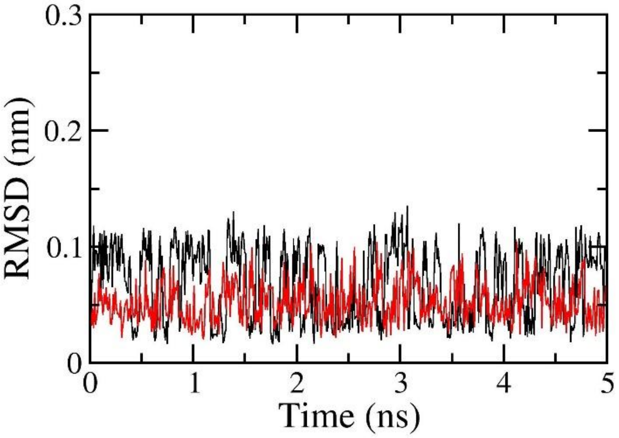 |
| 6 | 7L8I | 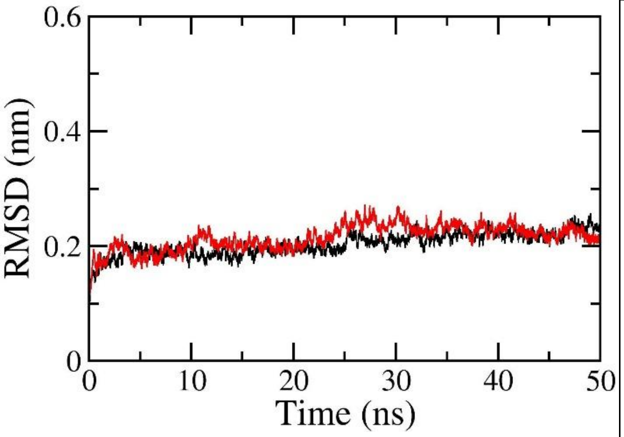 | 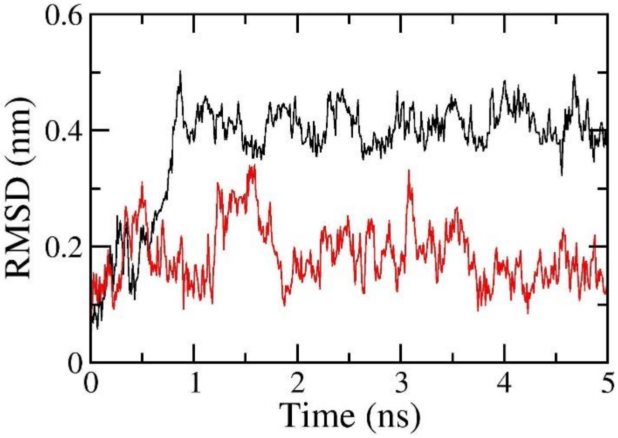 |

| | | | |
|---|---|---|---|
| 7 | 7LDL | 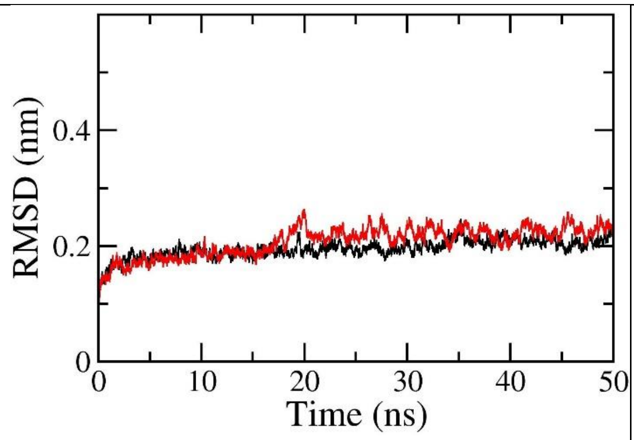 | 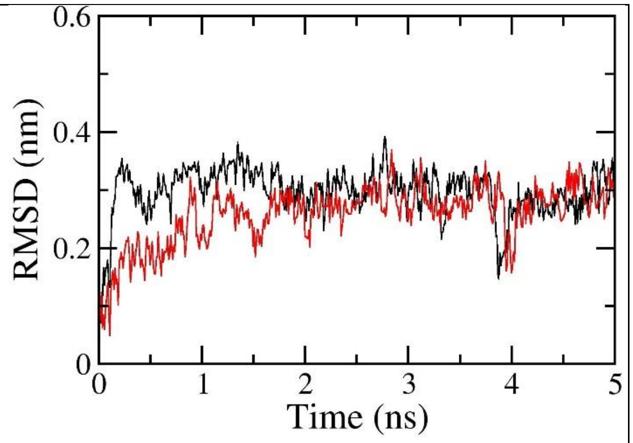 |
| 8 | 7NG3 | 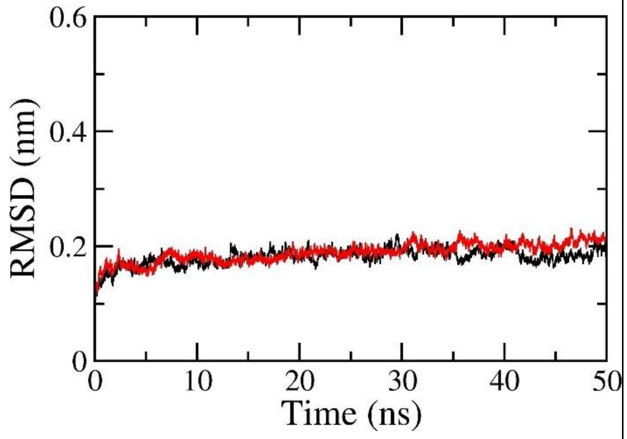 | 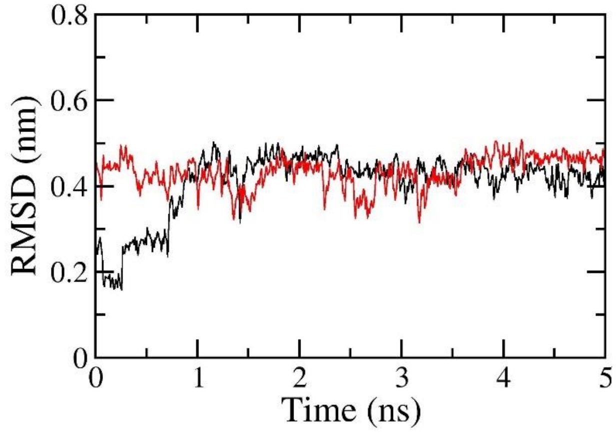 |
| 9 | 7CBT | 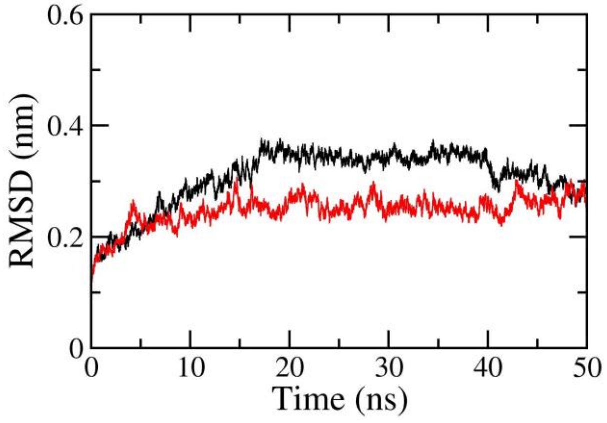 | 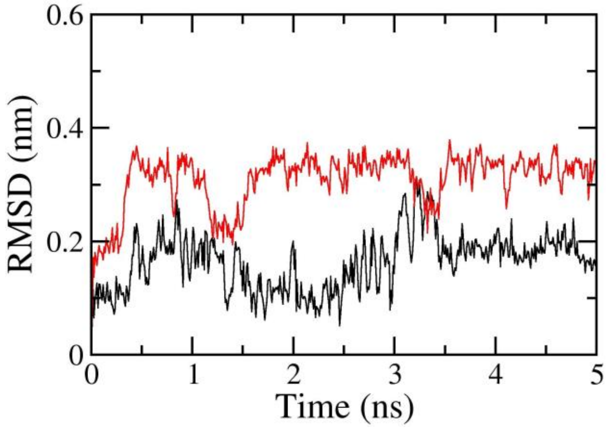 |

| | | | |
|---|---|---|---|
| 10 | 8A4T | 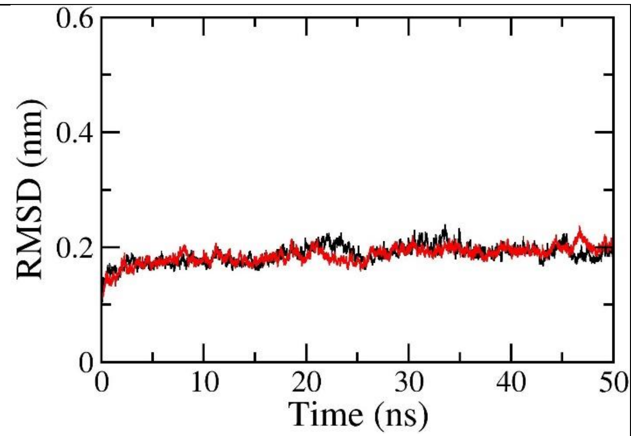0.6
0.4
0.2
0
RMSD (nm)
0
10
20
30
40
50
Time (ns) | 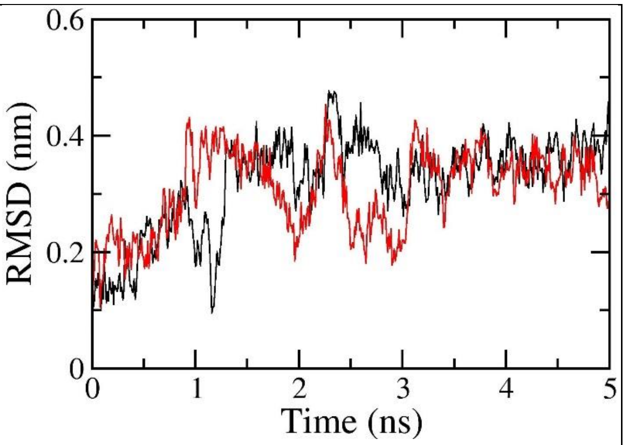0.6
0.4
0.2
0
RMSD (nm)
0
1
2
3
4
5
Time (ns) |

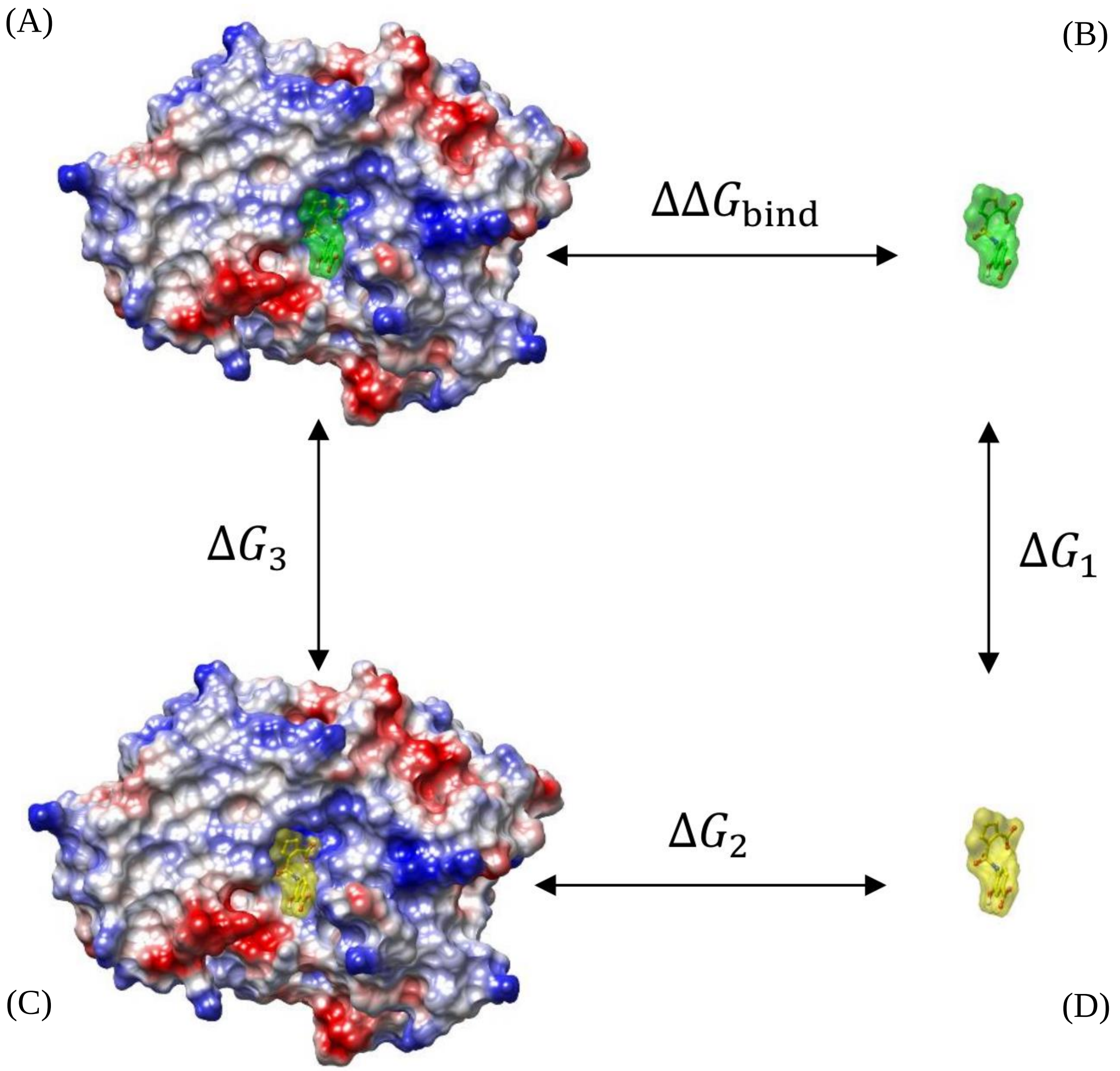


**Figure S1**. Thermodynamics diagram of FEP calculations. (A) full-interaction state of a ligand with surrounding protein and solvation; (B) full-interaction state of a ligand with surrounding solvation; (C) non-interaction state of a ligand with surrounding protein and solvation; (D) non-interaction state of a ligand with surrounding solvation.

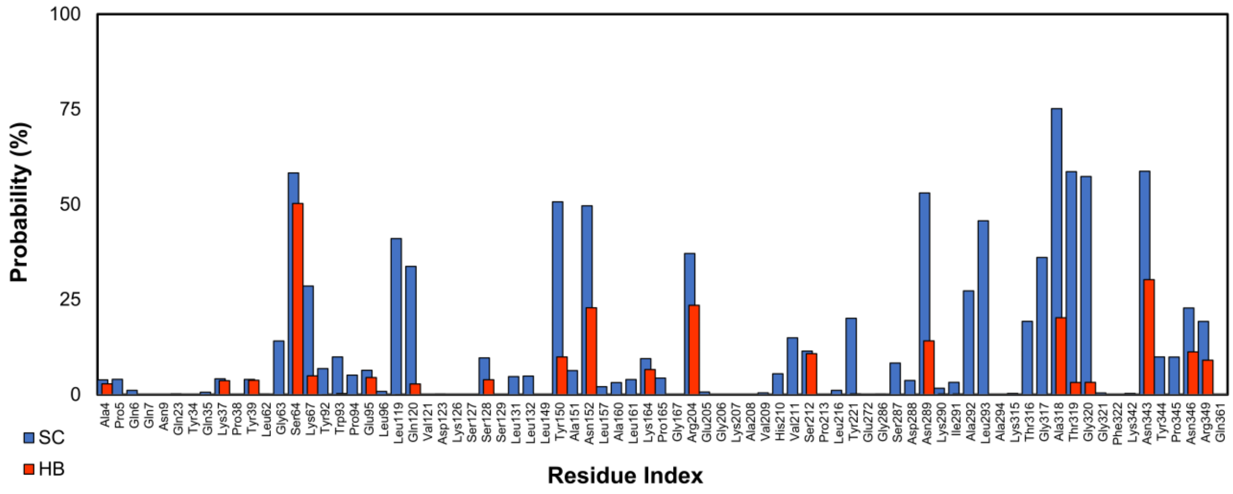

**Figure S2.** SC and HB Contacts between AmpC Residues and Inhibitors. The values were computed over the interval 25-50 ns of MD simulations.

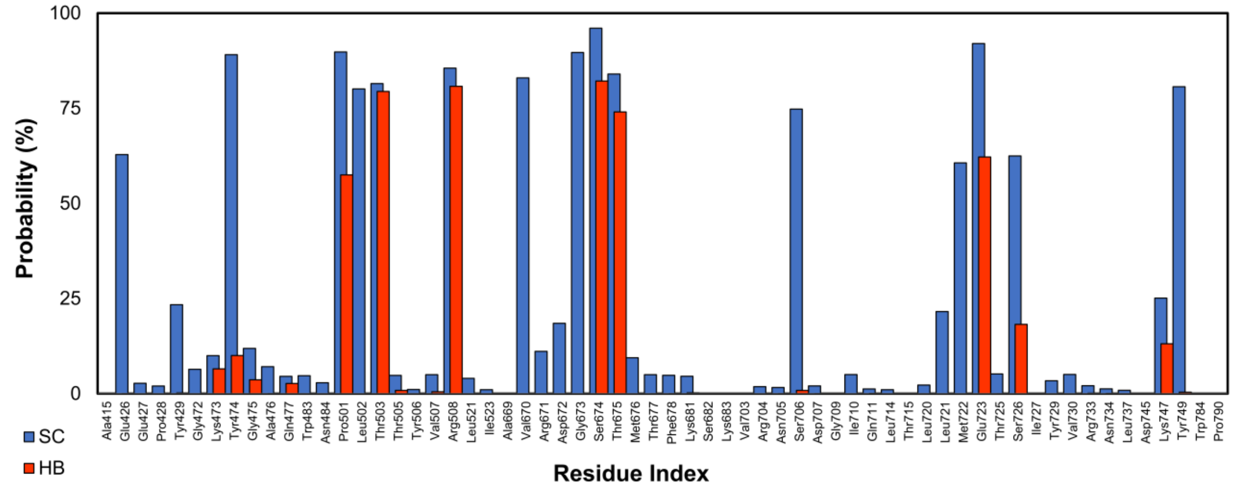

**Figure S3.** SC and HB Contacts between GluK1 Residues and Inhibitors. The values were computed over the interval 25-50 ns of MD simulations.

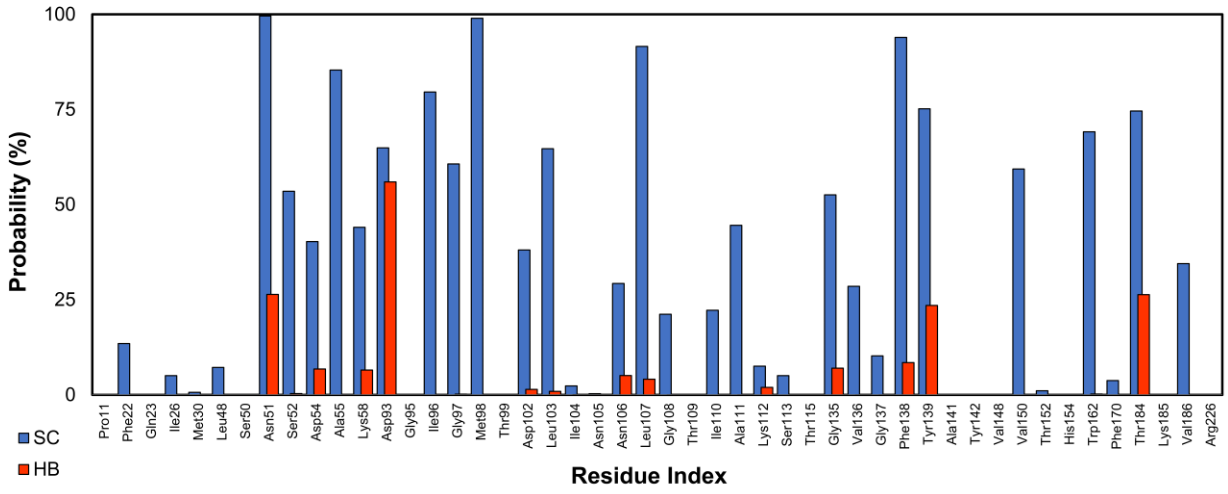


**Figure S4.** SC and HB Contacts between Hsp90 Residues and Inhibitors. The values were computed over the interval 25-50 ns of MD simulations.

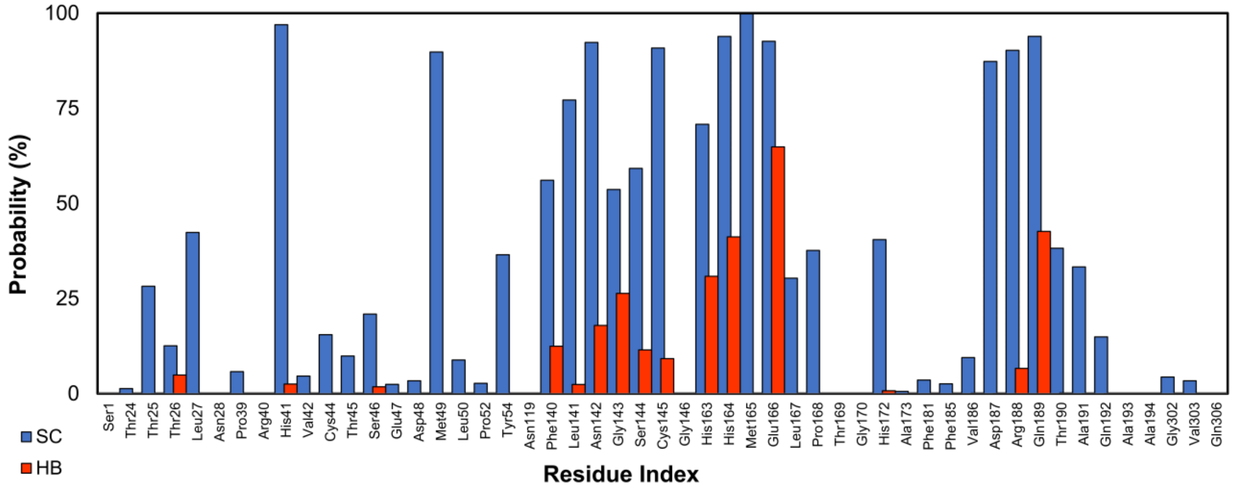


**Figure S5.** SC and HB Contacts between SARS-CoV-2 Mpro Residues and Inhibitors. The values were computed over the interval 25-50 ns of MD simulations.

**Table S6.** Calculated versus Experimental Binding Affinities between AmpC, GluK1, Hsp90, SARS-CoV-2 Mpro and its inhibitors

| N⁰ | PDB ID | PDB ID | $\Delta G_{cou}$ | $\Delta G_{vdW}$ | $\Delta G_{FEP}$ | $\Delta G_{EXP}$[b] |
|---|---|---|---|---|---|---|
| 1 | AmpC | 1XGJ | -2.41 | -5.10 | −7.50 ± 1.74 | -8.24 |
| 2 | GluK1 | 3FV1 | -21.90 | -0.78 | −22.68 ± 0.24 | -12.77 |
| 3 | Hsp90 | 3K97 | -2.74 | -8.66 | −11.40 ± 0.91 | -10.98 |
| 4 | SARS-CoV-2 Mpro | 6M2N | -0.67 | -5.83 | −6.51 ± 0.64 | -8.25 |

[b]The experimental binding free energies were computed based on the inhibition constant $k_{\mathrm{i}}$. The computed error was obtained using the bootstrapping method.

The unit is kcal mol$^{-1}$.

**Table S7.** All-atom RMSD of AmpC, GluK1, Hsp90, SARS-CoV-2 Mpro + ligand and ligand in solution systems.

| N⁰ | PDB ID | Complex in Solution | Ligand in Solution |
|---|---|---|---|
| **1** | **1XGJ** | 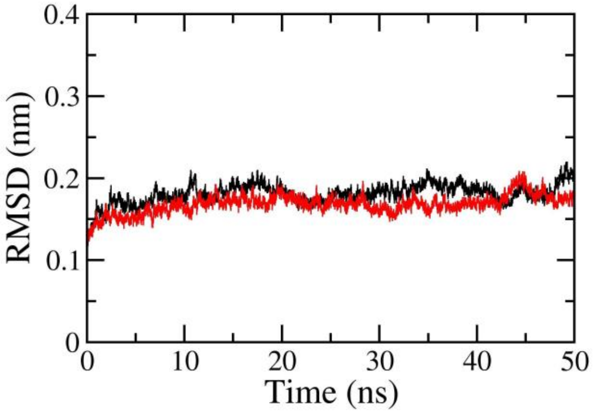<br> | 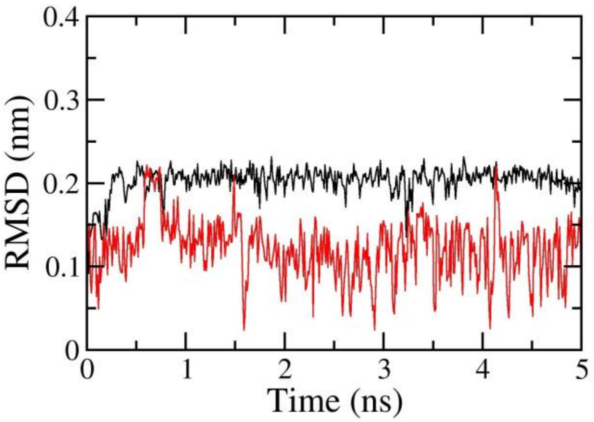<br> |
| **2** | **3FV1** | 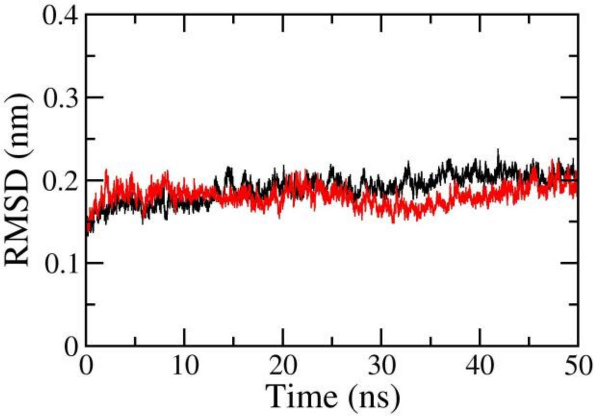<br> | 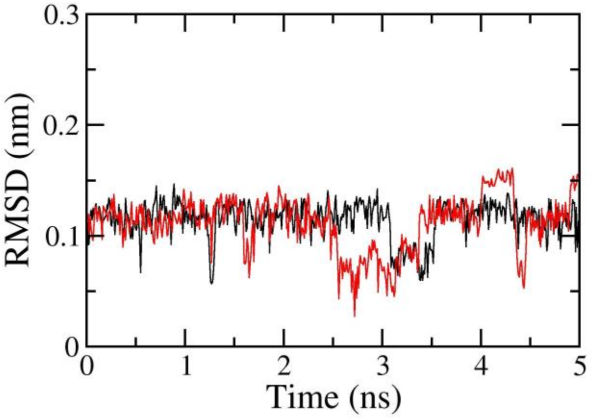<br> |

| | | | |
|---|---|---|---|
| 3 | 3K97 | 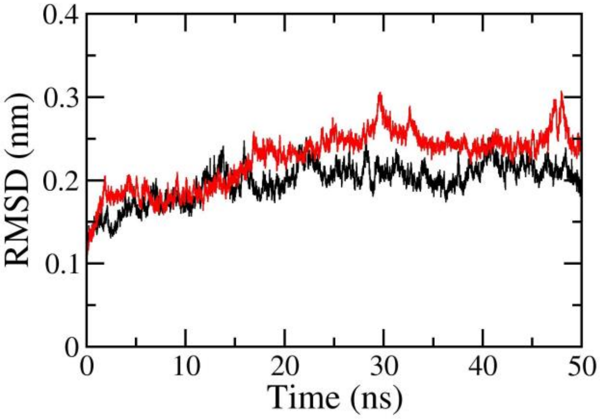 | 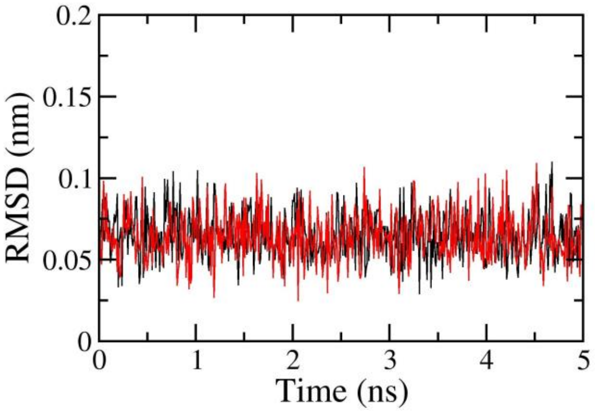 |
| 4 | 6M2N | 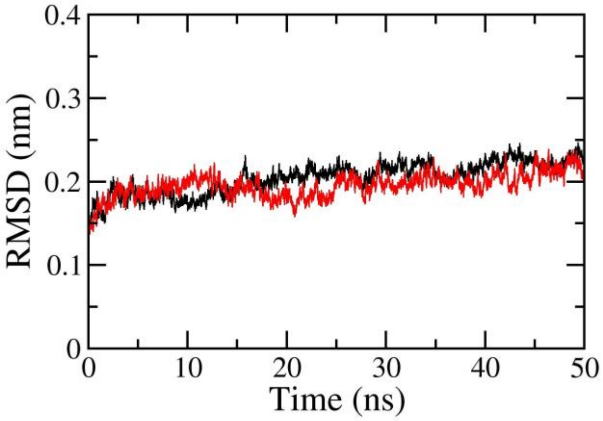 | 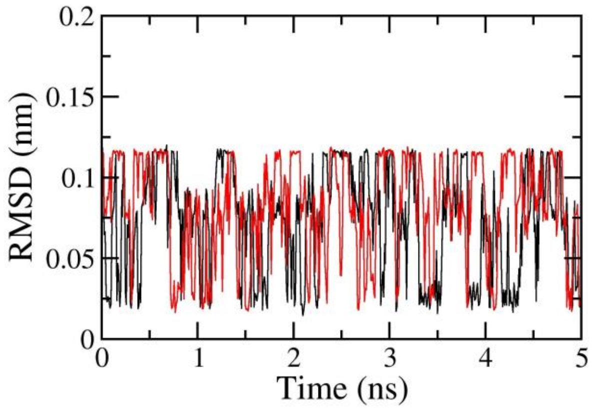 |

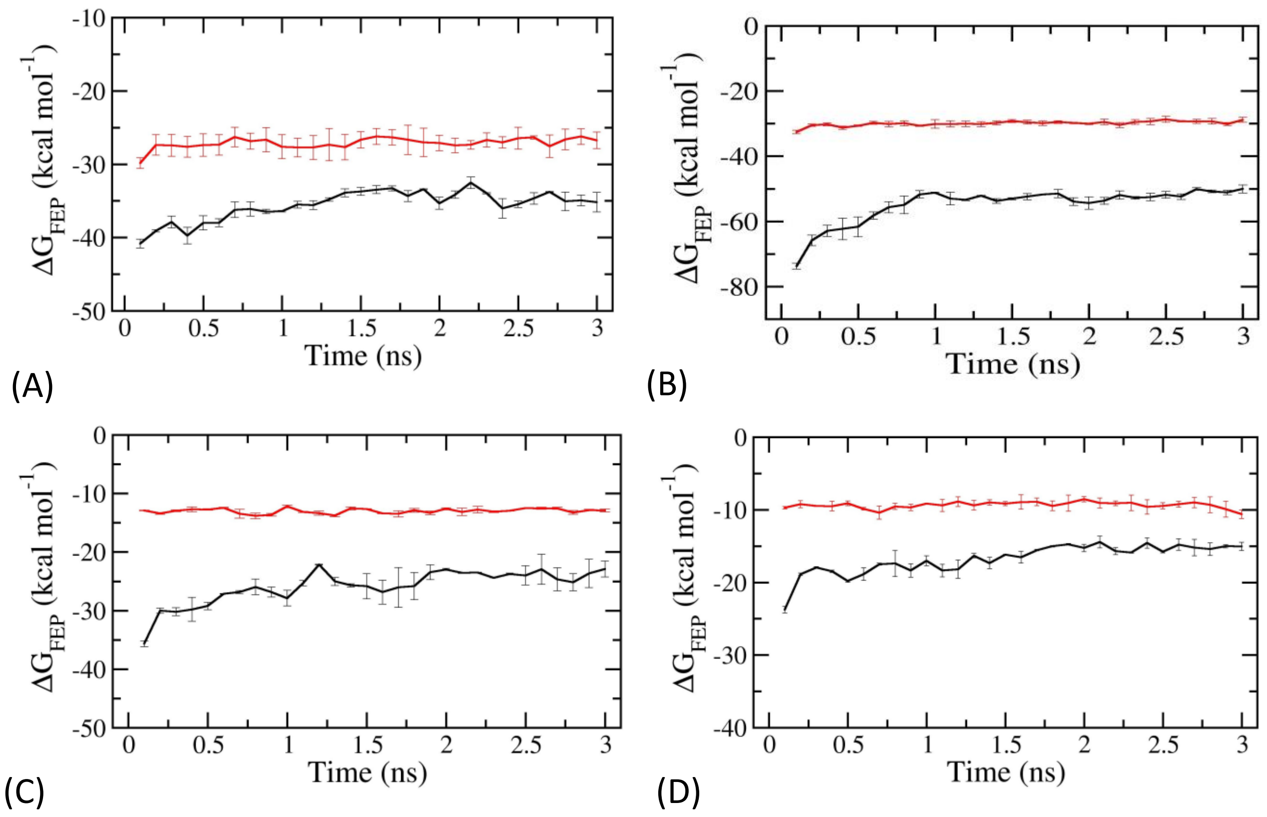


**Figure S6.** Time dependence of binding free energies for solvated complexes: (A) AmpC *(1XGJ)*, (B) GluK1 *(3FV1)*, Hsp90 *(3K97)*, SARS-CoV-2 Mpro *(6M2N*). The values collected in the equilibrium region were used to calculate $\Delta G_{FEP}$. The error bars show the root mean square deviations.
